# Confinement, Jamming, and Adhesion in Cancer Cells Dissociating from a Collectively Invading Strand

**DOI:** 10.1103/prxlife.3.013012

**Published:** 2025-02-25

**Authors:** Wei Wang, Robert A. Law, Emiliano Perez Ipiña, Konstantinos Konstantopoulos, Brian A. Camley

**Affiliations:** 1 Department of Physics & Astronomy, Johns Hopkins University, Baltimore, Maryland 21218, USA; 2 Department of Chemical and Biomolecular Engineering, Johns Hopkins University, Baltimore, Maryland 21218, USA; 3 Institute for NanoBioTechnology, Johns Hopkins University, Baltimore, Maryland 21218, USA; 4 Department of Biomedical Engineering, Johns Hopkins University, Baltimore, Maryland 21218, USA; 5 Department of Oncology, Johns Hopkins University, Baltimore, Maryland 21205, USA; 6 Department of Biophysics, Johns Hopkins University, Baltimore, Maryland 21218, USA

## Abstract

When cells in a primary tumor work together to invade into nearby tissue, this can lead to cell dissociations—cancer cells breaking off from the invading front—leading to metastasis. What controls the dissociation of cells and whether they break off singly or in small groups? Can this be determined by cell-cell adhesion or chemotactic cues given to cells? We develop a physical model for this question, based on experiments that mimic aspects of cancer cell invasion using microfluidic devices with microchannels of different widths. Experimentally, most dissociation events (“ruptures”) involve single cells breaking off, but we observe some ruptures of large groups (~20 cells) in wider channels. The rupture probability is nearly independent of channel width. We recapitulate the experimental results with a phase-field cell motility model by introducing three different cell states (follower, guided, and high-motility “leader” cells) based on their spatial position. These leader cells may explain why single-cell rupture is the universal most probable outcome. Our simulation results show that cell-channel adhesion is necessary for cells in narrow channels to invade, and strong cell-cell adhesion leads to fewer but larger ruptures. Chemotaxis also influences the rupture behavior: Strong chemotaxis strength leads to larger and faster ruptures. Finally, we study the relationship between biological jamming transitions and cell dissociations. Our results suggest unjamming is necessary but not sufficient to create ruptures.

## I. INTRODUCTION

Collective cell migration shows up in many developmental processes, like wound healing [1], embryogenesis [2,3], and tumor growth and invasion [4,5]. Compared to single-cell migration, collective migration is often more persistent since cells are more coordinated due to their mutual communications and interactions [5,6]. The ability of cells to rearrange fluidly in collective migration may be controlled by an unjamming transition [7–10] shown in dense biological tissues, which is similar to the well-known solid-liquid phase transition of materials [7]. This jamming-unjamming transition has been extensively studied using different theoretical models [7–11], which predict that cell tissues can be fluidized by increasing cell motility and deformability [7,8,10].

Collective cell migration *in vivo* often occurs within confined spaces [12–14]. For example, during cancer invasion, the primary tumor extends a strand of cells into surrounding tissues, resulting in cells being confined, followed by the detachment of a single cell or a group of cells from the strand. Cancer metastasis is a complex process that requires cancer cells first to detach from the invading strand of the primary tumor either individually or as small clusters and then migrate through adjacent tissue, circulate in the vasculature, and finally survive and proliferate in distant organs [5]. Intravital microscopy studies reveal that tumor cells preferentially migrate along channel-like tracks created by various anatomical structures *in vivo* [15]. The widths of these tracks range from highly confining (⩽3μm) to larger than the individual tumor cells [16,17]. Cancer cells that break away from the original tumor can migrate through interstitial tissues, enter the bloodstream, attach to the vascular endothelium, and cross it to infiltrate the target tissues [18]. Whether cells detach as single cells or clusters is a key controlling factor for cancer progression. Though clusters of tumor cells (usually 2–50 cells) circulating in the bloodstream are rather rare, they can drastically increase metastatic potential compared to single-cell seeding [5,6], dramatically increasing the harms from the cancer.

**FIG. 1.**
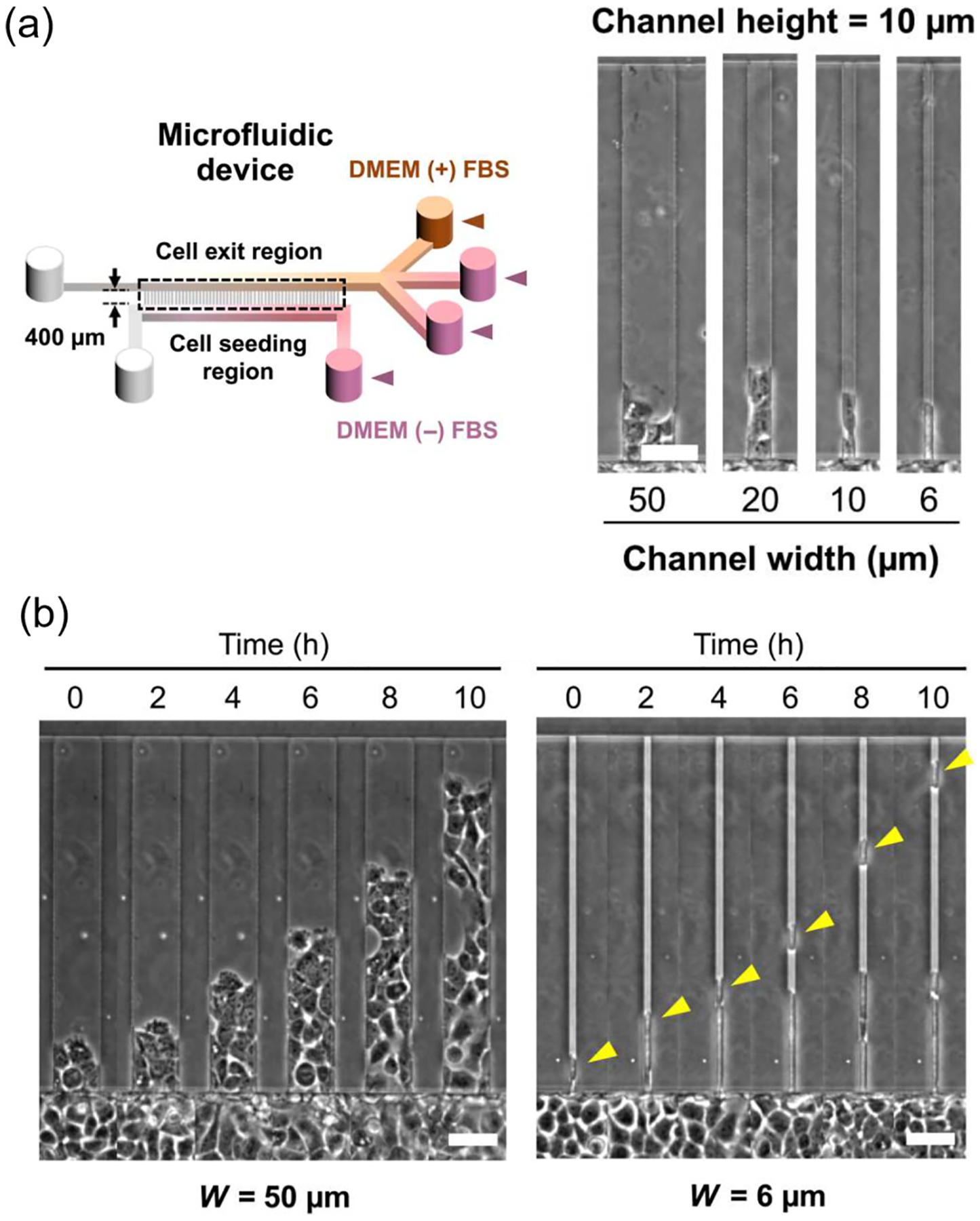
Experimental setup. (a) Schematic of microfluidic device with microchannels of prescribed dimensions and phase-contrast images of microchannels of different widths. Cells were cultured in Dulbecco’s modified Eagle’s medium (DMEM). (b) Representative phase-contrast snapshots of invading human A431 epidermoid carcinoma cells confined in 50- and 6-μm microchannels. Yellow arrowheads indicate an ongoing single-cell dissociation. Cells are exposed to a gradient of fetal bovine serum (FBS); FBS concentration increases in the +y direction. Gradients are established between the wells with FBS, labeled DMEM (+) FBS, which have serum-containing DMEM (20% FBS with 1% penicillin-streptomycin) and the wells without FBS, labeled DMEM (−) FBS, which have serum-free DMEM (containing only 1% penicillin-streptomycin). Scale bars, 50μm. Reprinted from Ref. [19]. Channel height from the substrate is 10 microns.

In this work, we study how single cells and clusters of cells dissociate from an invading front of a tumor under different degrees of geometric confinement. We use the approach we developed in Ref. [19] to study invasion and dissociation *in vitro*, with cells induced to invade into a narrow microchannel by a gradient of serum (Fig. 1). The narrow microchannel is designed to mimic the confinement that cells encounter *in vivo* as they migrate through the extracellular matrix, adjacent tissues, or the vasculature mentioned above. In these experiments, we see one or several cells as a cluster detach from an invading stream in a microchannel of controllable width [19]. We have observed that the majority of dissociation events (“ruptures”) are single-cell ruptures, but in wide channels rupture of larger clusters is more common. Interestingly, the timescale of rupture appears to be independent of channel width in the experiments. We construct a model of this dissociation process by describing the cells as deformable crawling objects using a phase-field model [7,20]. This model allows us to describe the shape of each individual cell, which is key to unjamming [7–10], while allowing us to model cells detaching from one another. By incorporating different cell states and cell-wall adhesion into the standard phase-field model, we find that our model recapitulates the cluster-size distribution and survival probability observed in experiments, but this requires the presence of leader cells and a decrease in intercellular adhesion under confinement. Subsequently, we investigate the impact of four critical parameters in our model—cell-cell adhesion strength, cell-wall adhesion strength, chemotaxis strength, and the number of leader cells—on the rupture behavior of invading monolayers in confinement. In particular, we find that increasing cell-cell adhesion makes dissociation slower but also increases the average size of the dissociating cluster. Leader cell formation probability controls the balance between single-cell and multiple-cell dissociations. Furthermore, we explore the relationship between cell dissociation behavior and the unjamming transition, finding that unjamming is necessary but not sufficient to create ruptures.

## II. EXPERIMENTAL RESULTS

We begin with a focus on our recent experiments using human A431 epidermoid carcinoma cells in microchannels [19], where confluent cell monolayers follow a gradient of chemoattractant (fetal bovine serum, FBS) to enter the microchannels. These experiments revealed details of the bio-chemical mechanism of cell dissociation from collectively migrating strands in confinement [19]. Figure 1(a) shows experimental snapshots for different microchannel widths. We can observe that cells are entering microchannels from the seeding region (the wide region with cells at the bottom) and following the chemical gradient. As the cells crawl through the microchannels toward the exit region on the opposite side, we observe that single cells or clusters of several cells can dissociate from the main bulk of the cell collective inside the microchannels. The detached cells then leave the bulk, crawling independently, and enter the exit region. We show an example of a single-cell dissociation in a 6-micron channel and a dissociation of a large cluster in Fig. 1(b).

**FIG. 2.**
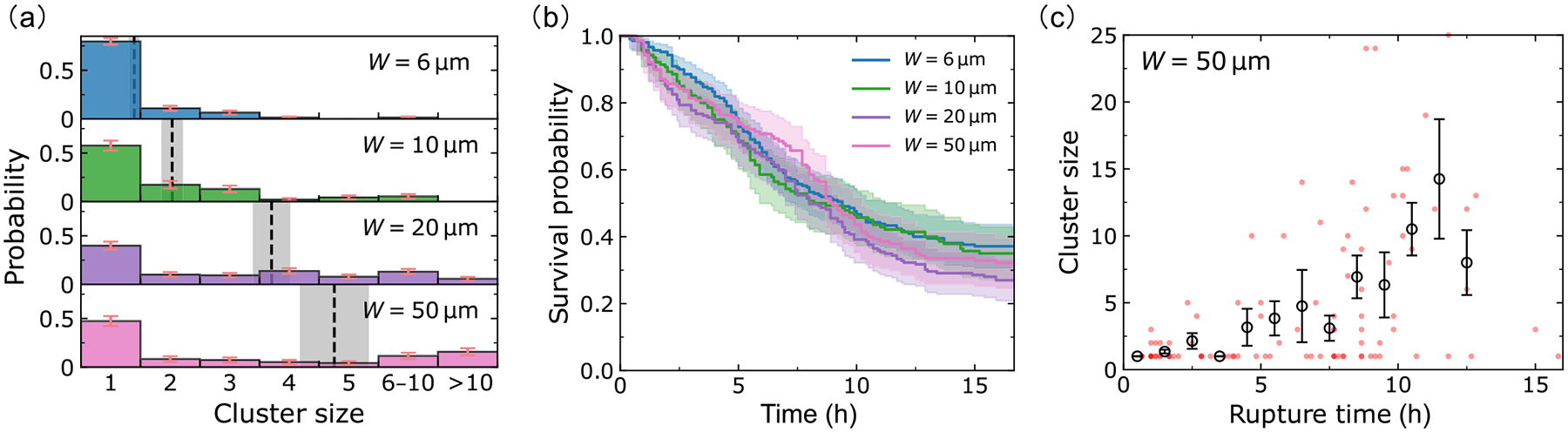
Analysis of experimental dissociation events. (a) Dissociation cluster size distributions for W=6-,10-,20-,50-μm microchannels, based on 137, 92, 138, and 95 dissociation events, respectively. The dashed lines and gray areas denote the average cluster sizes A=1.4, 2.0, 3.7, 4.8, and the associated standard errors of the mean (mean ± SE). Note that we are using cluster size categories 6–10 and >10 to group together similar-sized large clusters; full histograms are shown in Fig. 12(a). Panel (b) shows the survival curves for different channel widths extracted by Kaplan-Meier survival analysis [21]. The shaded areas represent the 95% confidence intervals; the final survival probabilities are $K_{s}=73/210(35\%)$, 48/140 (34%), 51/189 (27%), 45/140 (32%), respectively; the denominators of $K_{s}$ represent the number of assays in the experiment. (c) Scatter plot in the (rupture time, cluster size) phase plane. Data points (red) are plotted with transparency; darker points indicate ruptures occurring in more than one assay with the same cluster size and rupture time. The circles represent the average cluster sizes within each time bin (1 h), and the error bars indicate the corresponding standard errors. Figures for all channel widths are given in supplementary Fig. 12(b).

We reanalyze experiments of invasion in different channel widths first presented in Ref. [19], measuring the sizes of clusters breaking off and the times of cluster ruptures. We plot the distributions of cluster size as a function of microchannel width in Fig. 2(a). The mean cluster size A, plotted as dashed lines in Fig. 2(a), increases with channel width W. We see that across all the channel widths, though cluster size increases, the most common outcome is a single-cell dissociation. The probability of having a n-cell rupture decreases sharply with increasing n. However, in wide channels (20 and 50μm), we do see a good number of larger ruptures [Fig. 2(a)]. In fact, in the 50-micron channel, observing clusters of >10 cells is the second most probable category. For full histograms without categorization, see Appendix F.

How quickly do these ruptures happen? We characterize the timescale for the first rupture to happen by measuring the survival probability S(t), defined as the probability that the monolayer in a microchannel has not ruptured at time t. By comparing S(t) curves for different channel widths, we can see how fast ruptures happen in these channels, and the probability of any eventual rupture. Figure 2(b) shows the survival curves (probability of no rupture in a channel) and the corresponding error intervals obtained by standard Kaplan-Meier survival analysis [21]. By intuition, we would expect that larger channels would have more ruptures and ruptures occur sooner because more cells enter the larger channels—meaning more possible cells to dissociate. Surprisingly, the survival curves are—to the level of our statistical uncertainties—independent of channel width. We also see that the survival probability saturates to ~30% at long times, i.e., roughly ~70% of channels have a dissociation event within the experimental measurement.

To see the correlation between cluster size and rupture time, we make a scatter plot of cluster size as a function of the rupture time for the 50-micron channels; each dot in Fig. 2(c) corresponds to one dissociation event [analogous plots for smaller channel sizes shown in Fig. 12(b)]. Roughly 50% of the dissociations are single-cell ruptures; the dots corresponding to these are primarily in the bottom-left corner of Fig. 2(c), showing these single-cell events often occur at early times. On the other hand, larger ruptures are relatively infrequent and tend to occur at later times. This trend is further supported by binning the data into small time ranges, where we can see that the average cluster size—black circles in Fig. 2(c)—increases with rupture time. We believe the average cluster size increases with time in part simply because to have a rupture of n cells, we must wait for n cells to enter into the channel.

## III. MODEL

### A. Phase-field approach

To capture the complex shapes of cells, we use a multicell phase-field approach [22–25] to track the cell boundaries. We can model a cell interface by introducing an auxiliary phase field $\phi_{i}(r)$ for each cell i. This field smoothly varies from $\phi_{i}(r)=1$ inside the cell to $\phi_{i}(r)=0$ outside cell i. Consequently, the cell interface can be easily tracked at $\phi_{i}(r)=1/2$ [25].

To model the dynamics of the cell, we adopt a Newtonian-like advection scheme appropriate for an overdamped environment [7,26]:

(1) $$\frac{\partial \phi_{i}(r,t)}{\partial t}+P_{i}\cdot \nabla \phi_{i}+{\frac{\partial \phi_{i}}{\partial t}|}_{\text{adh}}=-\frac{1}{\gamma }\frac{\delta ℋ}{\delta \phi_{i}},$$

where the first two terms on the left-hand side describe cell i as being advected with a constant velocity $P_{i}$. We call $P_{i}$ the “polarity” of cell i—the velocity it would have in the absence of other cells pushing on it or forces arising from deformation of its boundary. The third term describes the evolution of $\phi_{i}$ due to cell-cell and cell-wall adhesion, which we will describe in detail later. On the right-hand side we introduce the functional derivative of a Hamiltonian ℋ [9,26,27]. This term tends to minimize ℋ [25]. γ is a friction coefficient so the whole Eq. (1) can be thought of as a force balance in an overdamped environment [28]—though see note.^1^ We include a Cahn-Hilliard term, an area constraint, and cell-cell and cell-wall exclusion in the Hamiltonian [7,26]:

(2) $$ℋ={ℋ}_{\text{CH}}+{ℋ}_{\text{area}}+{ℋ}_{\text{exclusion}}.$$

The first term in ℋ is the Cahn-Hilliard energy [9,26]

$${ℋ}_{\text{CH}}=\sum_{i}\int {\text{d}}^{2}r[\frac{\alpha }{4}\phi_{i}^{2}{\left(\phi_{i}-1\right)}^{2}+\frac{K}{2}{\left(\nabla \phi_{i}\right)}^{2}],$$

where $\phi_{i}^{2}{\left(\phi_{i}-1\right)}^{2}$ in the integrand is a double-well potential ensuring there exist two minima corresponding to the two phases ϕ(r)=0 and ϕ(r)=1; the second term ${\left(\nabla \phi_{i}\right)}^{2}$ sets the energy cost of forming a domain wall between the two phases. In general, the Cahn-Hilliard energy describes the interfacial tension [29] and controls the interfacial width $d=\sqrt{2K/\alpha }$ (see Appendix C). Also, we assume our cells resist changes in area, introducing a term [9,26]

$${ℋ}_{\text{area}}=\sum_{i}\lambda {\left(1-\int {\text{d}}^{2}r\frac{\phi_{i}^{2}}{\pi R^{2}}\right)}^{2},$$

which penalizes any deviation from the preferred area $\pi R^{2}$. We next introduce an energetic penalty to prevent cells from overlapping with other cells [26] or the walls of the microchannel,

$${ℋ}_{\text{exclusion}}=\sum_{i>j}\int {\text{d}}^{2}rg\phi_{i}^{2}\phi_{j}^{2}+\sum_{i}\int {\text{d}}^{2}rg_{\text{wall}}\phi_{i}^{2}\phi_{\text{wall}}^{2}.$$

The first term in ${ℋ}_{\text{exclusion}}$ describes cell-cell exclusion, which is a penalty for any overlapping between cells, since the integrand is only nonzero when both $\phi_{i}$ and $\phi_{j}$ are nonzero; the second term describes cell-wall exclusion in the same way as cell-cell exclusion—$\phi_{\text{wall}}(r)$ is another auxiliary field introduced to depict the geometric confinement—$\phi_{\text{wall}}=0$ inside the channel so cells can exist (no exclusion), and $\phi_{\text{wall}}=1$ for regions where cells are not allowed to enter.

The term $\partial \phi_{i}/{\partial t|}_{\text{adh}}$ in Eq. (1) models the effect of cell-cell and cell-wall adhesion,

$${\frac{\partial \phi_{i}}{\partial t}|}_{\text{adh}}=\sum_{j}\omega f(\nabla \phi_{j})\cdot \nabla \phi_{i}+\kappa f(\nabla \phi_{\text{wall}})\cdot \nabla \phi_{i}.$$

The first term treats cell-cell adhesion with the advection-like term in Ref. [24] and describes the attractive interactions between interfaces of different cells. Note that ∇ϕ is a vector pointing normally into the cell described by $\phi .f(\zeta )=\zeta /\sqrt{1+\epsilon |\zeta {|}^{2}}$ is a function that saturates at large |ζ| to avoid numerical instability [24]. We have chosen this form over alternate implementations [23,30], as we have found it possible to resolve high cell-cell adhesions without numerical instability. The second term in $\partial \phi_{i}/{\partial t|}_{\text{adh}}$ models the cell-wall adhesion in the same way, with a strength κ. Cell-wall adhesion arises because all the surfaces of the channel are coated with adhesive fibronectin [19]; we do not treat cell-substrate adhesion explicitly here because all cells have roughly the same contact area with the bottom substrate.

Each cell in our model is assigned a polarity $P_{i}=p_{i}(\text{cos}\;\theta_{i},\text{sin}\;\theta_{i})$. It is important to note that $P_{i}$ does not represent the actual velocity of the cell. As mentioned earlier, polarity $P_{i}$ corresponds to the velocity that the cell would exhibit in the absence of any interactions with other cells or obstacles, i.e., considering only the first two terms in Eq. (1). The actual velocity of each cell is the velocity once all the other effects have been applied. We have set the default value of the magnitude $p_{i}$ to $p_{0}=13.3\;\mu \text{m}/\text{h}$—at the typical experimental cell velocity scale, though slower than cells that typically break off [19]. For the direction $\theta_{i}$, we assume that cells tend to align their polarity to the direction $\theta_{0}$ of the chemical gradient but there are still fluctuations that would lead to rotational diffusion,

(3) $$\frac{\partial \theta_{i}}{\partial t}=-k_{\theta }(\theta_{i}-\theta_{0})+\sqrt{2D_{r}}\xi_{i}(t),$$

where $\xi_{i}(t)$ is a Gaussian white noise satisfying $\langle \xi_{i}(t)\rangle =0$, and $\left\langle \xi_{i}(t)\xi_{j}(t^{\prime })\rangle =\delta_{ij}\delta (t-t^{\prime }\right)$, and the preferred direction $\theta_{0}$ is determined by the chemotactic cue. The first term on the right-hand side drives $\theta_{i}$ to relax to $\theta_{0}$ on a timescale of $\sim 1/k_{\theta }$. In the absence of chemotaxis ($k_{\theta }=0$), the angle $\theta_{i}$ will simply diffuse with angular diffusion coefficient $D_{r}$. This representation of cell polarity is a very simplified one, and could be extended by models of Rho GTPase dynamics [20,31] or stochastic protrusion dynamics [32].

### B. Cell states—Chemotaxis and leader cells

Above, we described cells as following a chemoattractant gradient. However, not all of the cells are likely to be able to sense the chemical signal—since the chemoattractant concentration will be diluted as it reaches the bottom of the microchannel. Rather than explicitly modeling chemoattractant dynamics, we make a simple assumption and distinguish the cells that are in the seeding region (reservoir at the bottom of Fig. 1) and cells that are in the microchannel. We assume cells located in the seeding region are not guided by the chemical gradient—cells in the seeding region are just followers of those guided cells moving along the chemical gradient in the channel.

In addition, there is well-established evidence that in collective migrations like wound healing [1], and cancer metastasis [4,5,33], cells invade the free space under the apparent guidance of some highly motile “leader” at their leading edge [12,34,35], which can be crucial in collective invasion [36,37]. These leader cells can invade the free surface more easily than other cells and coordinate their motion with their followers. Within cancerous collectives, leader cells may be keratin-14 positive cells which can either activate at or move to the leading edge and guide collective migration in tumor invasion [38–40]. Many effects of cell leadership have been proposed, including creating a path or coordinating with follower cells [33–35]. Instead, we take a relatively simplified assumption, which is that the only phenotypic difference between leader cells and other cells is that leader cells will have a larger self-propulsion strength $p_{i}$ than other cells.

**FIG. 3.**
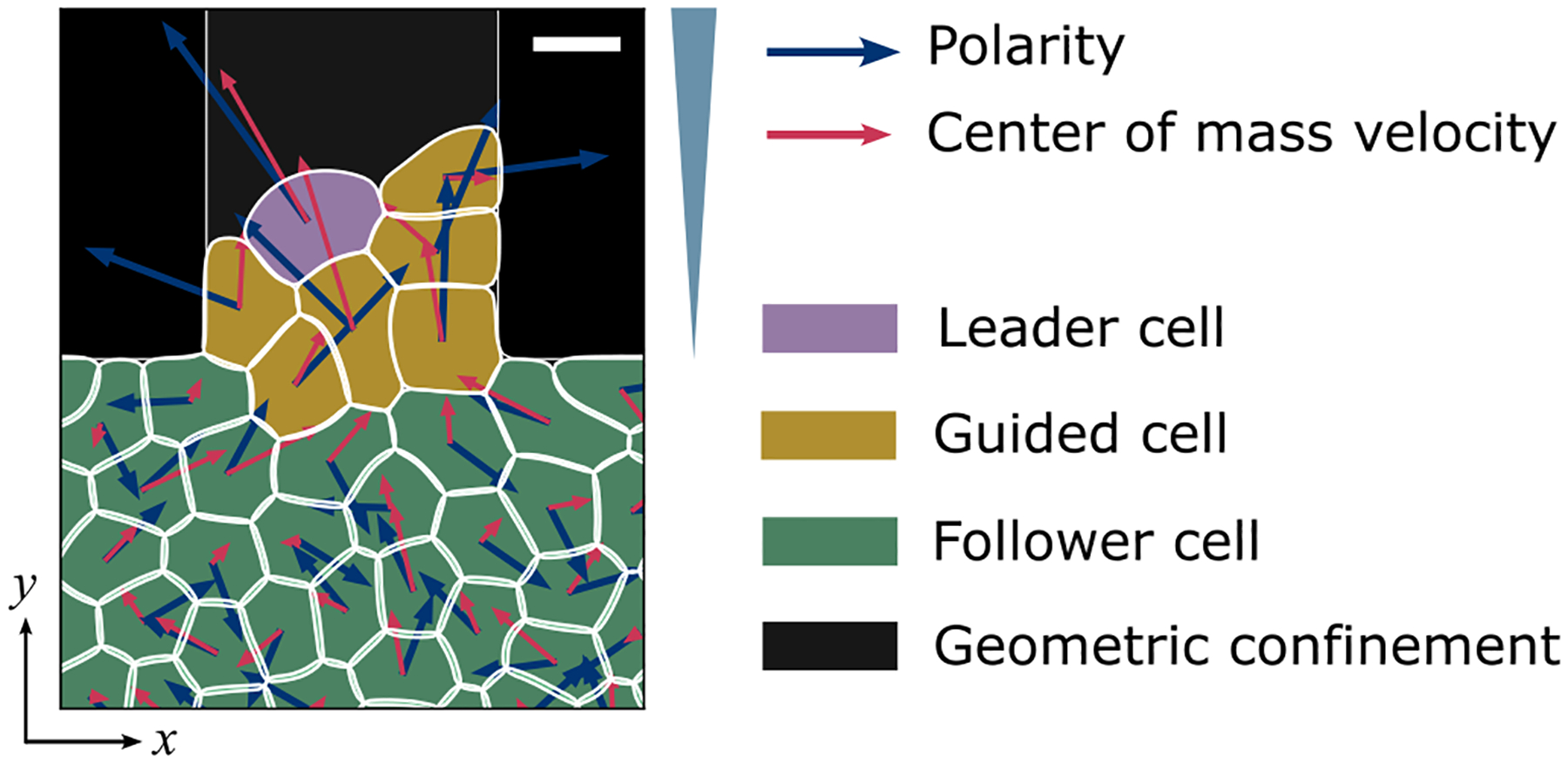
Schematic of the model. For each cell, the blue arrow represents its polarity $P_{i}$, and the red arrow shows its center-of-mass velocity $v_{i}^{\text{c.m.}}$; chemoattractant concentration increases in the +y direction. Scale bar, 15μm.

We illustrate the three cell states—follower (green), guided (yellow), and leader (purple)—described in our model in Fig. 3. The blue arrows represent the polarity $P_{i}$ and the red arrows indicate the center-of-mass velocity $v_{i}^{\text{c.m.}}$ (see Appendix D). Followers (green) are cells with chemotaxis strength $k_{\theta }=0$ and polarity magnitude $p_{\text{follower}}=p_{0}$. Guided cells (yellow) sense the chemoattractants and typically exhibit higher velocities than followers—modeling chemokinesis and chemotaxis [41]—i.e., guided cells have a nonzero $k_{\theta }$ and larger magnitude of polarity $p_{\text{guided}}=2p_{0}$. At the time a cell reaches the front, it has a probability of becoming a leader cell is dependent on its contact length with other cells:

(4) $$P_{i}^{\text{leader}}=\text{max}(1-L_{i}/L_{c},0),$$

where $L_{i}$ is the contact length of the ith cell with its neighbors and $L_{c}$ is a characteristic length. In practice, we count the number of cell-cell contact points $n_{i}$ for each cell, hence $P_{i}^{\text{leader}}=\text{max}(1-n_{i}/n_{c},0)$, where $n_{c}$ is the characteristic number of contact points (see Appendix D for details). Equation (4) reflects the idea that cell-cell contact inhibits leadership—similar to the proposal in Ref. [12]. This differs slightly from other models that choose leader cells randomly near the front [34,35].

The only distinction between leader cells and guided cells is that leader cells have stronger self-propulsion $p_{\text{leader}}=3p_{0}$. With this value, leader cells that dissociate will have speeds of up to $\sim 3p_{0}\approx 40\;\mu \text{m}/\text{h}$ while nonleader cells that dissociate will have speeds of up to $\sim 2p_{0}\approx 26\;\mu \text{m}/\text{h}$. These are both within the broad range of typical dissociating cell speeds (up to ~50μm/h) observed in 6-micron channels [19]. Experimentally in 6-micron channels, the difference between the mean speed of a cell that dissociates and a cell that is left behind after dissociating is roughly ~20μm/h (Fig. 1 D of Ref. [19]). Our model results are also roughly consistent with this observation, with typical y velocities of cells inside the invading front of 0–20μm/h, and escaping single cells able to reach up to ~26–40μm/h, depending on whether the dissociating cell is a leader.

Currently, the best model we find that can fit the experimental data (as shown in Fig. 2) is composed of these three types of cells: Cells near the channel entrance and inside the channel are identified as guided cells (labeled yellow in Fig. 3), and cells in the chamber are the followers (green) following the guided cells, while leader cells (purple) are tip cells at the invading front. Alternate models without leader cells are shown in Appendix A.

## IV. MODEL RESULTS

### A. Matching with experimental results

**FIG. 4.**
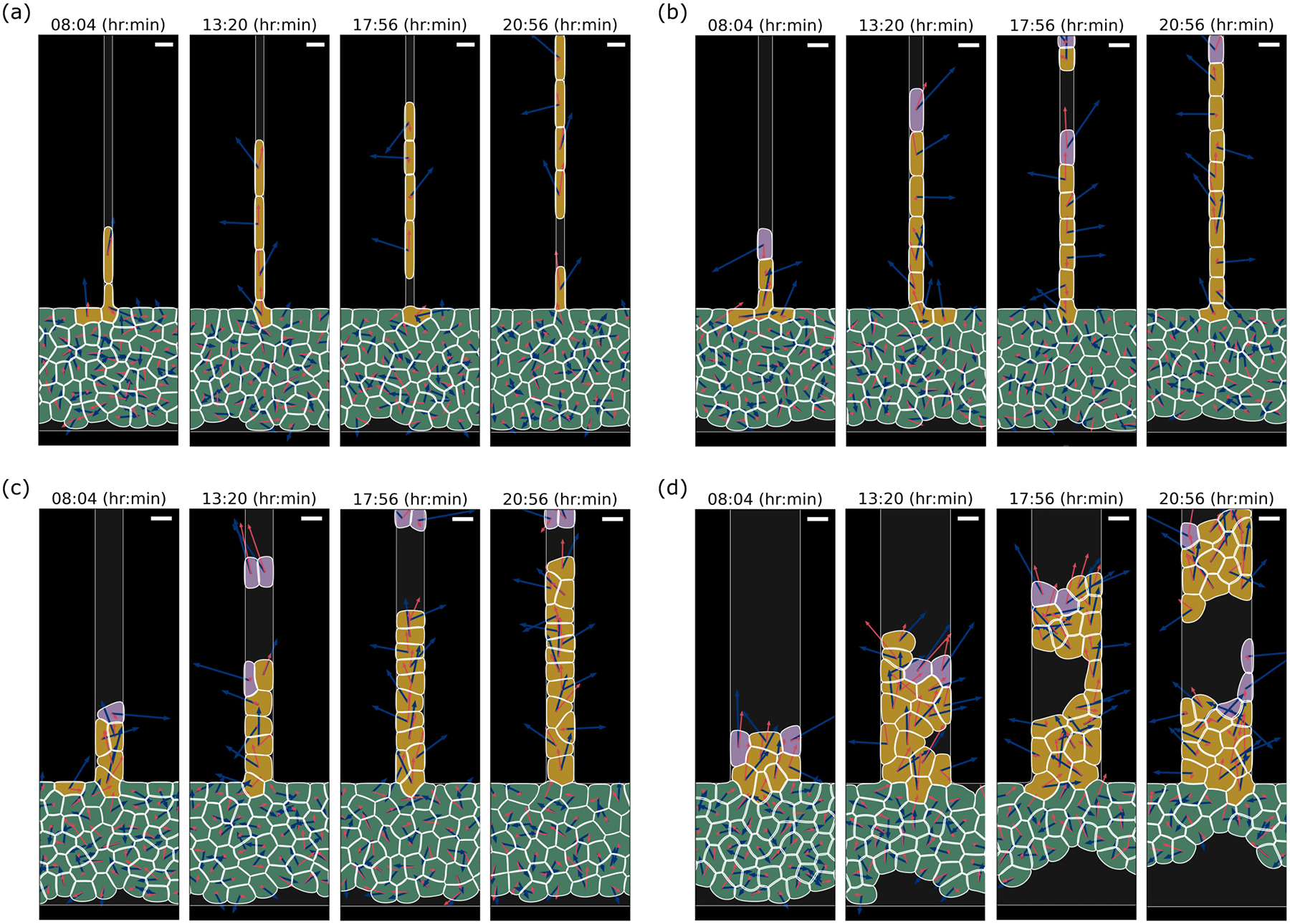
Evolution of rupture for simulations of cells in microchannels with different widths. Panels (a)–(d) correspond to W=6, 10, 20, and 50μm, respectively. There are chemical gradients along the microchannels (+y direction) driving cells to move upwards. Scale bars, 15μm.

**FIG. 5.**
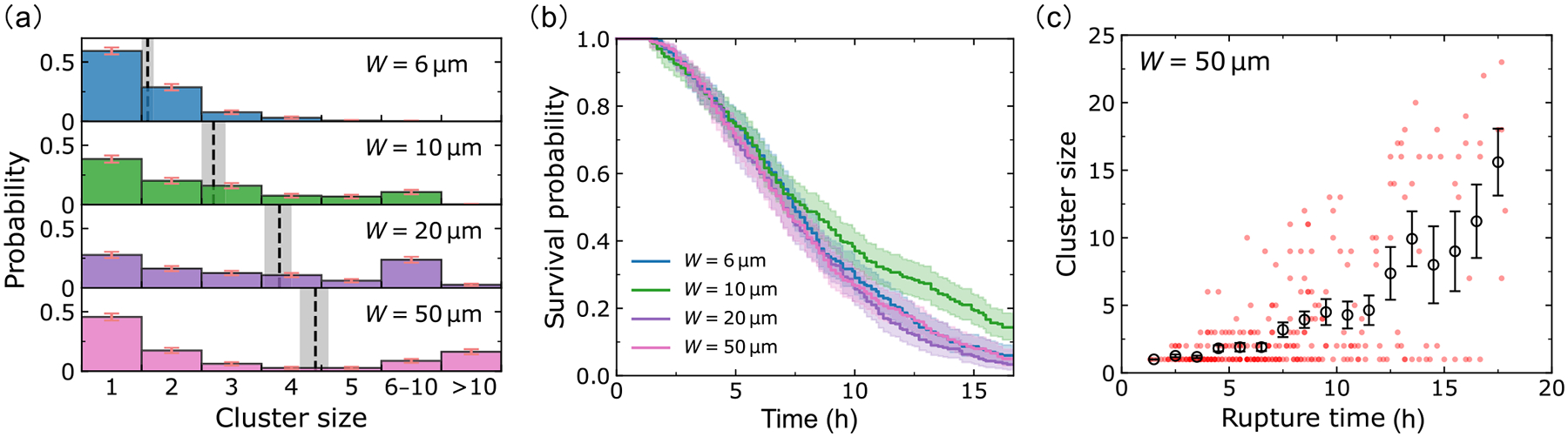
Simulation results to match experimental data. We adjust $\omega =0.7\omega_{0}$ for the two narrowest channels, $\omega =0.8\omega_{0}$ for 20-μm channels, and $\omega =\omega_{0}$ for 50-μm channels. (a) Cluster-size distributions for W=6-, 10-, 20-, 20-μm microchannels, based on 284, 257, 292, 287 dissociation events, respectively. The corresponding average cluster sizes (dashed lines; mean ± SE) are A=1.6, 2.7, 3.8, 4.4. The complete histograms are shown in Fig. 13(a). Panel (b) shows the survival probabilities for different channel widths; the final survival probabilities are $K_{s}=16/300$, 43/300, 8/300, 13/300, respectively. Panel (c) shows the (rupture time, cluster size) phase plane scatter plot. The circles represent the average cluster sizes within each time bin (1 h), and the error bars indicate the corresponding standard errors. Figures for all channel widths are given in Fig. 13(b). We conduct 300 independent simulations for each microchannel width W with $k_{\theta }=1.4k_{\theta }^{0}$ in all channels; $\omega_{0}$ and $k_{\theta }^{0}$ represent the reference values for adhesion and chemotaxis strength in the simulation.

We simulate our phase-field model for a maximum duration of 18 h, after an initial equilibration time where cells relax to more physical shapes (see Appendix D). Since our focus is on the first dissociation events and their sizes, the simulations are terminated as soon as the first detachment occurs—except for those we show for illustration. Figure 4 shows the simulation snapshots for W=6-,10-,20-,50-μm microchannels (see also supplementary movies 1–4 [42]). We observe both single-cell and cluster ruptures. Figure 5 shows statistics on rupture size and times for our simulations, analogous to our experimental results in Fig. 2. We have been able to recapitulate several key factors of the experiment. Specifically, our model reproduces (i) the predominance of single-cell ruptures, (ii) the occurrence of larger ruptures in wider channels, (iii) the independence of survival curves from microchannel width, and (iv) the increase of average cluster size with rupture time.

When varying the channel width, we include the possibility that cell tension and adhesion dynamics change in confinement. Earlier experiments showed that in a narrow confinement, contractility increases, which leads to E-cadherin disengagement. In addition, cell-cell adhesions are more dynamic in narrow confinement [19,43,44]. We thus expect that the effective cell-cell adhesion should decrease as cells are placed into narrow confinement—but there is no direct quantitative measurement of adhesion strength as a function of confinement. This unavoidably requires some degree of tuning of parameters as a function of channel width. We take the simplest route we think is reasonable and change only the cell-cell adhesion parameter. To match the survival probability with experiments in Fig. 2(b), we have to increase cell-cell adhesion strength ω from $0.7\omega_{0}$ to $\omega_{0}$ for channel widths from 6-50μm, where $\omega_{0}$ is the reference value of adhesion strength in our model (see Table I for details). This is a relatively small tuning of adhesion strength but is sufficient to ensure that the survival curves do not differ strongly as a function of channel width [Fig. 5(b)]. If we do not make this assumption, and instead assume that all channels have the same value of cell-cell adhesion strength $\omega_{0}$, then we find that the 10-micron channel has slower rupture than other channels [Figs. 10(b) and 10(f)]. This is because cells in the 10-micron channel have a broader cell-cell contact line than the 6-micron channel—but in the 20-micron channel, multiple cells can enter at once, so ruptures do not require breaking a larger contact line (see Appendix B and Fig. 11).

The experimental cluster-size distributions [Fig. 2(a)] are well captured by our model [Fig. 5(a)]. We observe that single-cell ruptures are always predominant, though there is a population of larger ruptures in wide channels, leading to a mean rupture size that increases with channel width. We note that the single-cell ruptures being predominant depends on the presence of leader cells that are phenotypically different from other cells; we show how cluster statistics depend on the leader cell number in Sec. IV F, and show results without leader cells in Figs. 10(c) and 10(d).

We plot rupture cluster size as a function of rupture time in Figs. 5(c) and 13(b). Similarly to Fig. 2(c), we find that small ruptures dominate and usually happen at early times, and large ruptures are less probable and tend to happen at later times. Moreover, we see—consistent with the experiment—that the average cluster size increases with rupture time.

We note that the survival curves in Fig. 5(b) drop nearly to zero, which the experimental curves do not. We think this may reflect the evolution of parameters like the chemoattractant strength and cell-cell adhesion over time in our experiments and address this further in Sec. V.

Next, we systematically adjust several key parameters in the model to investigate their impact on the rupture behavior of the monolayers, including the cell-cell adhesion strength ω, cell-wall adhesion strength κ, chemotaxis strength $k_{\theta }$, and the probability for tip cells to become leader cells.

### B. Rough intuitive picture: Active escape over a barrier

Thinking about a chain of invading cells, such as shown in Fig. 4, we expect that rupture is not likely to happen if all cells are moving with consistent velocities. Differences in the self-propulsion from one cell to another lead to the cell centers moving apart from one another—increased strain. This difference in self-propulsion could arise because cell polarities point in different directions, e.g., due to the rotational noise $D_{r}$, or have different magnitudes, due to one cell having a different state from its neighbors. The difference in motilities between cells tends to work against forces keeping cells together—largely cell-cell adhesion. In this way, we think of rupture as being initiated by fluctuations of cell motility leading to the cells crossing an energy barrier [45,46]. For channels wider than a single cell can span, a dissociation event will start with the breakage of a single cell-cell junction, but this fracture will then have to evolve to cross the channel, as seen in, e.g., Refs. [47,48] and in Fig. 1(b) for the 50-micron channel. Our picture here is an oversimplification because there is not a single energy barrier, but a complex process involving rearrangement of cells and cell boundary deformations. Nonetheless, this rough picture makes some clear predictions. We would expect faster rupture if the cell motion is noisier, and more rupture if there is more variability in cell speeds—both increasing the tendency for cells to be pulled apart. We would expect less rupture if there is high cell-cell adhesion (increasing the barrier height)—and if the process of rupture is nucleated at the walls, then cell-wall adhesion could also influence the barrier. We test all of these ideas below.

### C. Cell-wall adhesion allows cells to invade into narrow channels

**FIG. 6.**
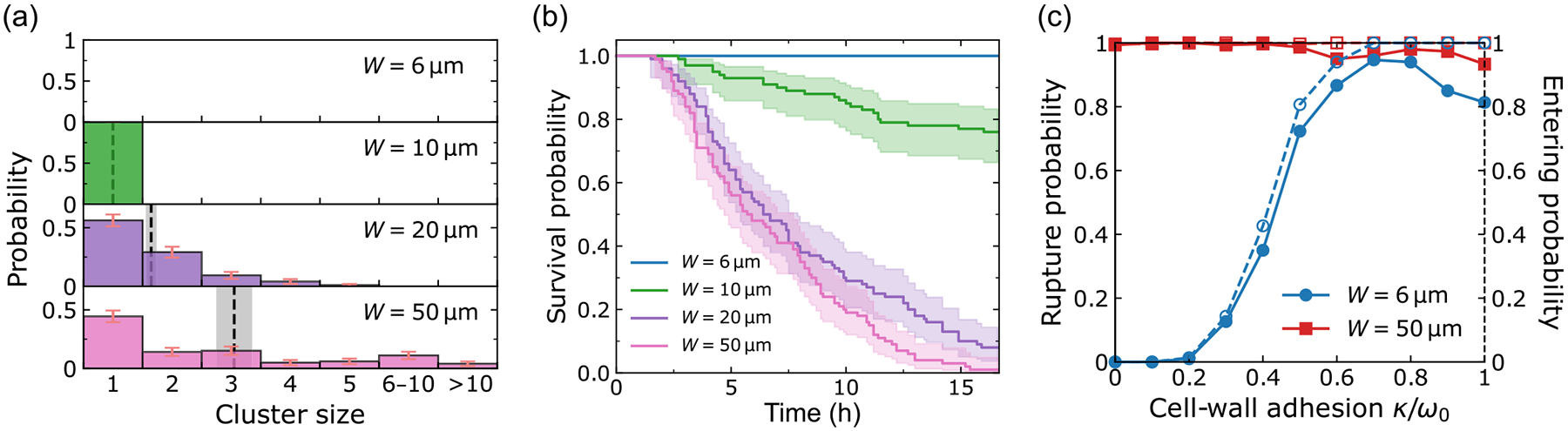
Cell-wall adhesion helps cells invade into narrow channels. Panels (a) and (b) are simulation results (100 independent simulations) when there is no cell-wall adhesion term. The corresponding average cluster sizes (dashed lines; mean ± SE) are A=0, 1.0, 1.6, 3.1, and the final survival probabilities are $K_{s}=100/100$, 76/100, 8/100, 1/100. The complete histograms are shown in Fig. 14(a). Panel (c) shows the rupture probability $1-K_{s}$ (solid lines) and the probability of cells entering the channel (dashed lines) as a function of cell-wall adhesion strength κ for narrow (6-μm) and wide (50-μm) microchannels; 300 independent simulations for each point.

Cell-wall adhesion is vital to observe ruptures in narrow channels. Figures 6(a) and 6(b) show the cluster-size distributions and survival curves when we turn this term off. The rupture behavior in wide channels (like 50-μm channels) is not immediately different in the absence of cell-wall adhesion, but ruptures are strongly suppressed in narrow (6- or 10-μm) channels—most dramatically, no ruptures occur in 6-μm microchannels. This is because, in this scenario, cells fail to enter the narrow channels (see supplementary movie 5 [42]). We change the cell-wall adhesion strength κ and see how this influences rupture in narrow (6-micron) and wide (50-micron) channels. As shown in Fig. 6(c), κ has little effect on 50-μm channels, but it can control the rupture probability (1 – final survival probability) in narrow channels. Rupture fraction in small channels increases rapidly with cell-wall adhesion and then saturates, consistent with the idea that cell-wall adhesion is helping cells overcome an energy barrier at the channel entrance [49]. The rupture fraction is low at small cell-wall adhesion because in these cases, few cells manage to enter the channel. We plot the probability of any cells entering the channel in Fig. 6(c) using dashed lines, and it closely tracks the rupture fraction—at small κ, if any cell enters the channel, then there will almost always be a dissociation. Interestingly, rupture probability in both 6- and 50-micron channels eventually decreases as cell-wall adhesion is increased. We believe this is because some ruptures require detachment from the wall as part of their dissociation process—e.g., the formation of the “notch” in the 50-micron channel simulation in Fig. 4(d) and supplementary movie 4 [42]. Cell-wall adhesion thus has two effects: some amount of cell-wall adhesion helps cells enter channels, but large cell-wall adhesion helps prevent cells from dissociating.

### D. Strong cell-cell adhesion leads to rarer but larger ruptures

**FIG. 7.**
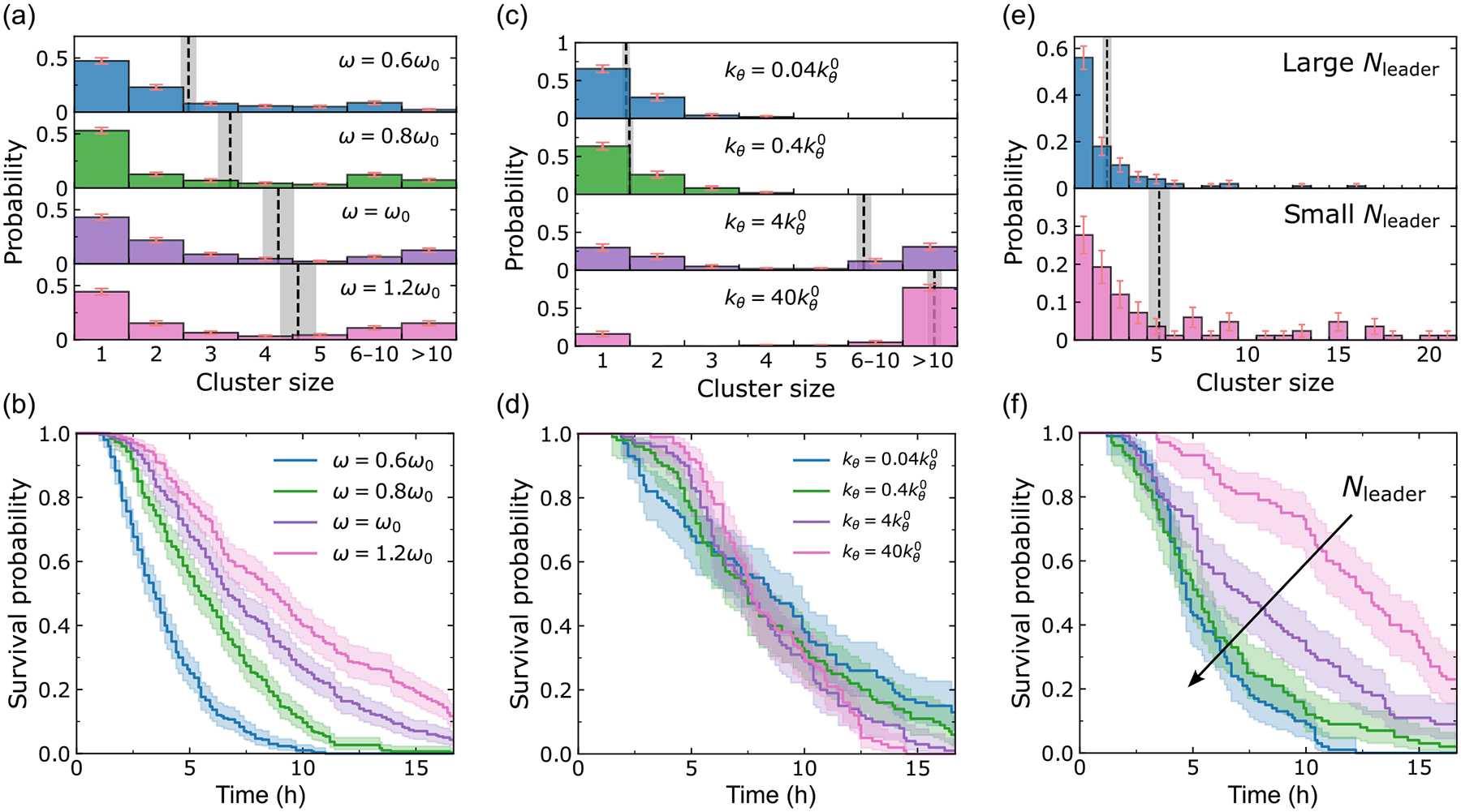
How intercellular adhesion, chemotaxis, and leader cells affect the dissociation behaviors. Panels (a) and (b) are simulation results for different cell-cell adhesion strength ω in W=50μm channels. The corresponding average cluster sizes (dashed lines; mean ± SE) are A=2.6, 3.4, 4.2, 4.6, and the final survival probabilities are $K_{s}=0$, 1/100, 4/100, 11/100. Panels (c) and (d) are simulation results for different chemotaxis strength $k_{\theta }$ in W=50μm microchannels. The corresponding average cluster sizes (dashed lines; mean ± SE) are A=1.4, 1.5, 6.9, 13.4, and the final survival probabilities are $K_{s}=12/100$, 6/100, 1/100, 0/100. Panels (e) and (f) are simulation results when the probability of leader cell formation [Eq. (4)] is altered by setting the characteristic number of contact points $n_{c}$ to $0.5n_{c}^{0}$, $n_{c}^{0}$, $2n_{c}^{0}$, and $10n_{c}^{0}$, where $n_{c}^{0}=256$ (see Appendix D). Larger $n_{c}$ corresponds to larger $N_{\text{leader}}$. The corresponding average cluster sizes (dashed lines; mean ± SE) are A=2.3, 5.2. The complete histograms for panels (a) and (c) are shown in Figs. 14(b) and 14(c).

Ruptures occur at cell-cell junctions for epithelial monolayers, and the intercellular adhesion complexes, such as different members of the cadherin family, can control the strength of tissues [47]. In our model, cell-cell adhesion strength ω is one of the most important parameters that prescribe the rupture behavior. Our intuition is that increasing cell-cell adhesion strength ω should increase the barrier to rupture and reduce the frequency of rupture events. Figures 7(a) and 7(b) show the simulation results for 50-μm microchannels. With the increase of cell-cell adhesion, the survival curve drops much more slowly, and the final survival rate rises a little—rupture is less common due to the strong connection between cells. This influence of cell-cell adhesion is consistent with our earlier experimental work, which found that in 6-micron channels, increasing the cell-cell adhesion by overexpressing E-cadherin led to suppressed rupture, and downregulation by shRNA led to increased rupture [19].

From the cluster-size distribution, we find there are more ruptures of larger clusters (>10 cells) happening in systems with strong cell-cell adhesion, and mean cluster sizes increase with increasing adhesion ω. In general, strong cell-cell adhesion leads to less rupture, but if we get a rupture, then it is more likely to be a large rupture. Our results show that the influence of changes in cell-cell adhesion on metastasis may be complex, and we would expect its effect on overall risk to depend on the relative importance of the rate and size of metastases, even neglecting any effect of cadherins on cell proliferation and survival.

### E. Strong chemotaxis strength leads to larger and faster ruptures

We expect that the rate of rupture could potentially be increased if the cells are noisier in their motility—i.e., if they are less uniformly guided by the presence of the chemoattractant gradient. This is in part controlled by the chemotaxis strength $k_{\theta }$.

To better understand the magnitude of $k_{\theta }$, we think about the dynamics of cell orientation θ for a single isolated cell, which follows Eq. (3). We take the preferred direction $\theta_{0}=0$ and then, by the properties of Ornstein-Uhlenbeck processes [50], the average is ⟨θ⟩=0, and the variance is $\langle \theta^{2}\rangle =D_{r}/k_{\theta }$. Cell chemotactic accuracy is often characterized by a “chemotactic index” CI=⟨cosθ⟩ (or related definitions) [51]; CI = −1 would be perfect chemorepulsion, CI = 0 is undirected migration, and CI = 1 is perfect, deterministic motion up the gradient. For our model, we can compute for an isolated cell $\text{CI}=\langle \text{cos}\;\theta \rangle =\text{exp}(-\langle \theta^{2}\rangle /2)=\text{exp}(-D_{r}/2k_{\theta })$, using the Gaussian distribution of θ. This gives us a way to interpret $k_{\theta }$. Our default value of $k_{\theta }=1.4k_{\theta }^{0}=0.014\;{\text{min}}^{-1}$ corresponds to a chemotactic index of ⟨cosθ⟩≈0.45. However, this estimate—which essentially assumes an isolated cell not in confinement—may not always reflect the chemotactic accuracy of cells in our full simulation. First, in our system, we have many cells—so the motion of their center of mass will be more accurate than any individual cell [51]. Second, cells in narrow channels are forced to have their velocity in the direction of the channel, so they will be more directional in confinement [13,52].

Figures 7(c) and 7(d) show the cluster-size distributions and the survival curves for different chemotaxis strengths $k_{\theta }$. These simulations are in 50-μm microchannels, and cell-cell adhesion strength is set to the default $\omega =\omega_{0}$. We vary $k_{\theta }$ across a large range ($0.04k_{\theta }^{0}-40k_{\theta }^{0}$, which corresponds to a CI range of roughly 0–1). From Fig. 7(c), we see that more ruptures of larger size happen at large $k_{\theta }$ when cell chemotaxis is more deterministic—cells move together as a large pack. In Fig. 7(d), when there is only very weak guidance (small $k_{\theta }$), when the cells can be polarized in almost any direction, not only toward the channel, we can see more early ruptures (survival curve drops earlier) but also see more channels that never have ruptures. By contrast, at very strong guidance (large $k_{\theta }$), the survival curves become steeper, indicating that a large chunk of cells move together and faster, and at $k_{\theta }=40k_{\theta }^{0}$ large clusters are the majority outcome.

### F. Leader cells drive single-cell rupture

Within our model, the key factor that controls whether cells have variable states is the probability for cells that reach the leading edge to become “metabolically active” leaders—with a high degree of self-propulsion, given by Eq. (4). We can increase the probability of cells becoming leader cells—and thus the average number of leader cells $N_{\text{leader}}$—by increasing the characteristic number of contact sites $n_{c}$, and vice versa.

Figure 7(e) shows the cluster-size distributions for large $N_{\text{leader}}$ and small $N_{\text{leader}}$, and Fig. 7(f) shows the survival curves for different $N_{\text{leader}}$ in 50μm channels, with $\omega =\omega_{0}$, and $k_{\theta }=1.4k_{\theta }^{0}$. The survival curves decrease faster with larger $N_{\text{leader}}$, which tells us that more leader cells lead to faster ruptures. Also, from Fig. 7(e) we can see that the leader cells increase the ratio of single-cell ruptures to all ruptures, which ensures the absolute predominance of single-cell ruptures shown in experiments. These leader cells, which have the largest velocity in the system and are located at the leading edge of the expanding monolayer, can dissociate from the main bulk more easily. Since leaders are relatively rare in our default parameters, this may explain the universal most probable outcome of single-cell rupture in all cluster-size distributions.

### G. Unjamming is necessary but not sufficient to create ruptures

A confluent monolayer can go from a jammed state to an unjammed state, allowing cells within the monolayer to more freely rearrange [53,54]. Experiments have shown that jamming is influenced by factors such as cell shape, cell motility, and cell-cell adhesion [54–56], and the spatiotemporal control of tissue states may represent a generic physical mechanism of embryonic morphogenesis [57]. Reference [7] shows that several different order parameters can capture this transition behavior. For instance, if a dimensionless quantity, called shape index, $q=\text{cell perimeter}/\sqrt{\text{cell area}}$, is larger than some critical value $q_{c}$ (Ref. [8] indicates $q_{c}\approx 3.81$), then the system is in a fluidlike unjammed state, where cells can squeeze between neighbors; if q is smaller than $q_{c}$, then the system is in a more solidlike jammed state, where cells are somewhat caged by their nearest neighbors. References [7,8] also show that a system in the solid state can be fluidized by increasing cell motility, cell deformability, or the preferred shape index of the tissue.

**FIG. 8.**
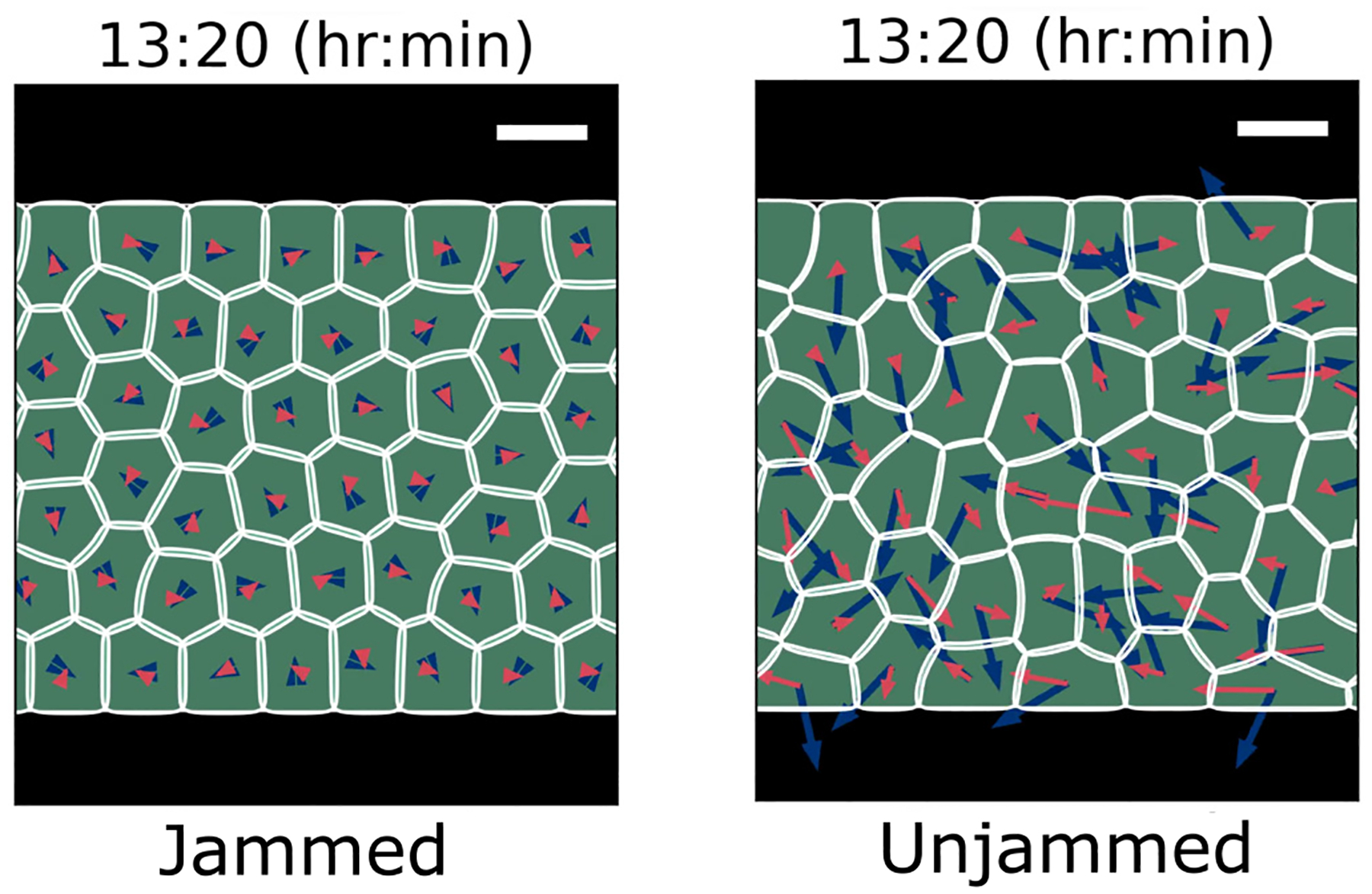
Simulation snapshots for typical systems in jammed (Pe = 0.14) and unjammed (Pe = 1.43) states at the same simulation time. Blue arrows represent the polarity $P_{i}$ and red arrows indicate the center-of-mass velocity $v_{i}^{\text{c.m.}}$. Scale bars, 15μm.

**FIG. 9.**
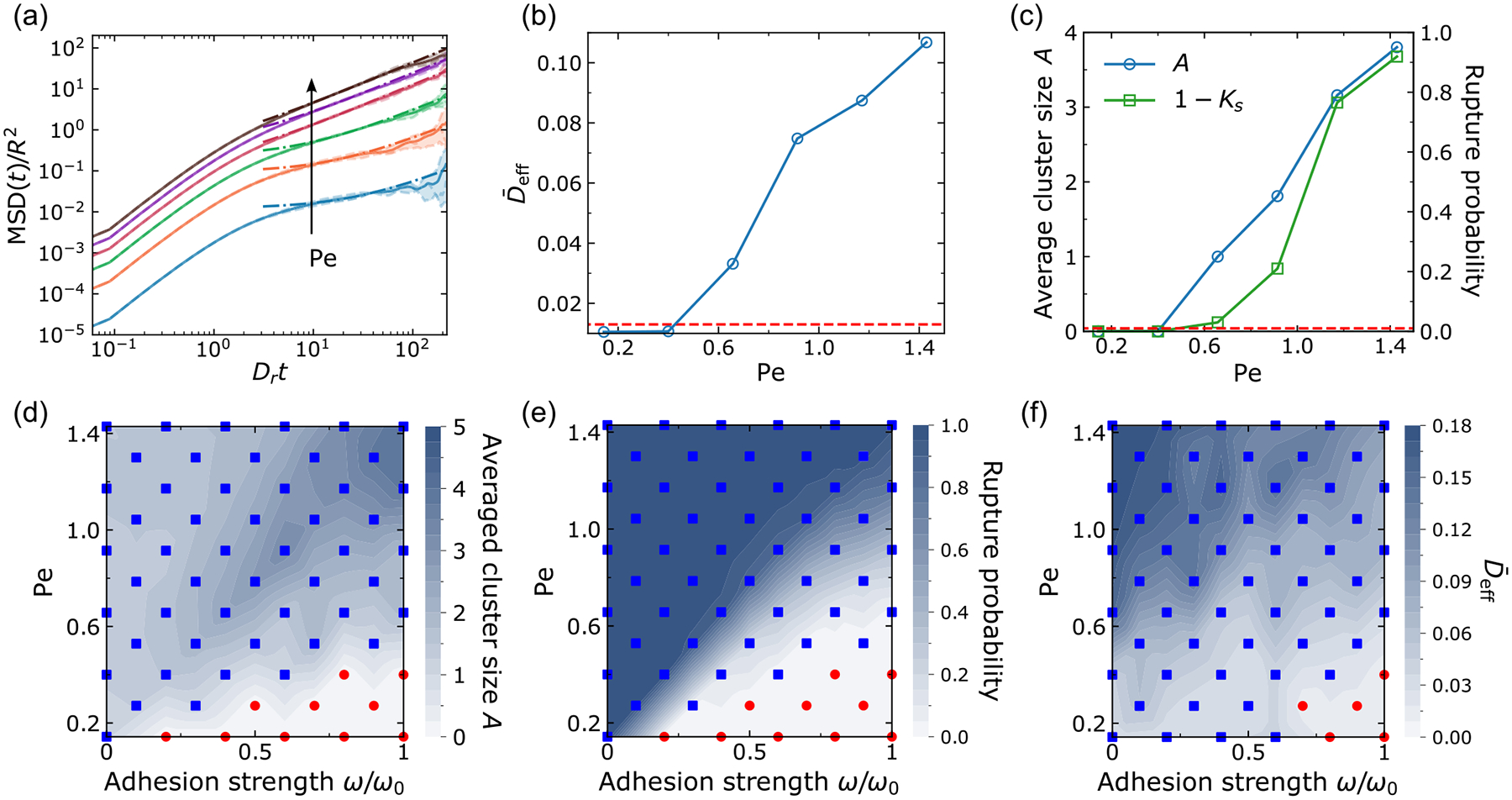
Unjamming and rupture. (a) Mean-squared displacement MSD(t) for different cell motility quantified by a dimensionless Péclet number $\text{Pe}≔p_{0}/(RD_{r})$. The different Pe shown in this panel are 0.14, 0.40, 0.66, 0.91, 1.17, and 1.43 (default); we have rescaled MSD and t to make them dimensionless; for each Péclet number, the dashed lines are results from three independent simulations, and the solid line is their average, and the dash-dotted lines are linear fit at late times where the effective diffusivity ${\overset{‾}{D}}_{\text{eff}}$ is extracted. (b) The effective diffusivity ${\overset{‾}{D}}_{\text{eff}}$ as an order parameter for solid-liquid transition. (c) Corresponding rupture transition of average cluster size (blue) and rupture probability (green). Panels (d) and (e) are phase diagrams for rupture transition. Red points indicate values equal to 0 and blue squares represent values greater than 0. (f) Phase diagram for jamming transition. Red points indicate values below the threshold value 0.012 and blue squares represent values above the threshold value. Three hundred independent simulations for each point in panels (c)–(f).

**FIG. 10.**
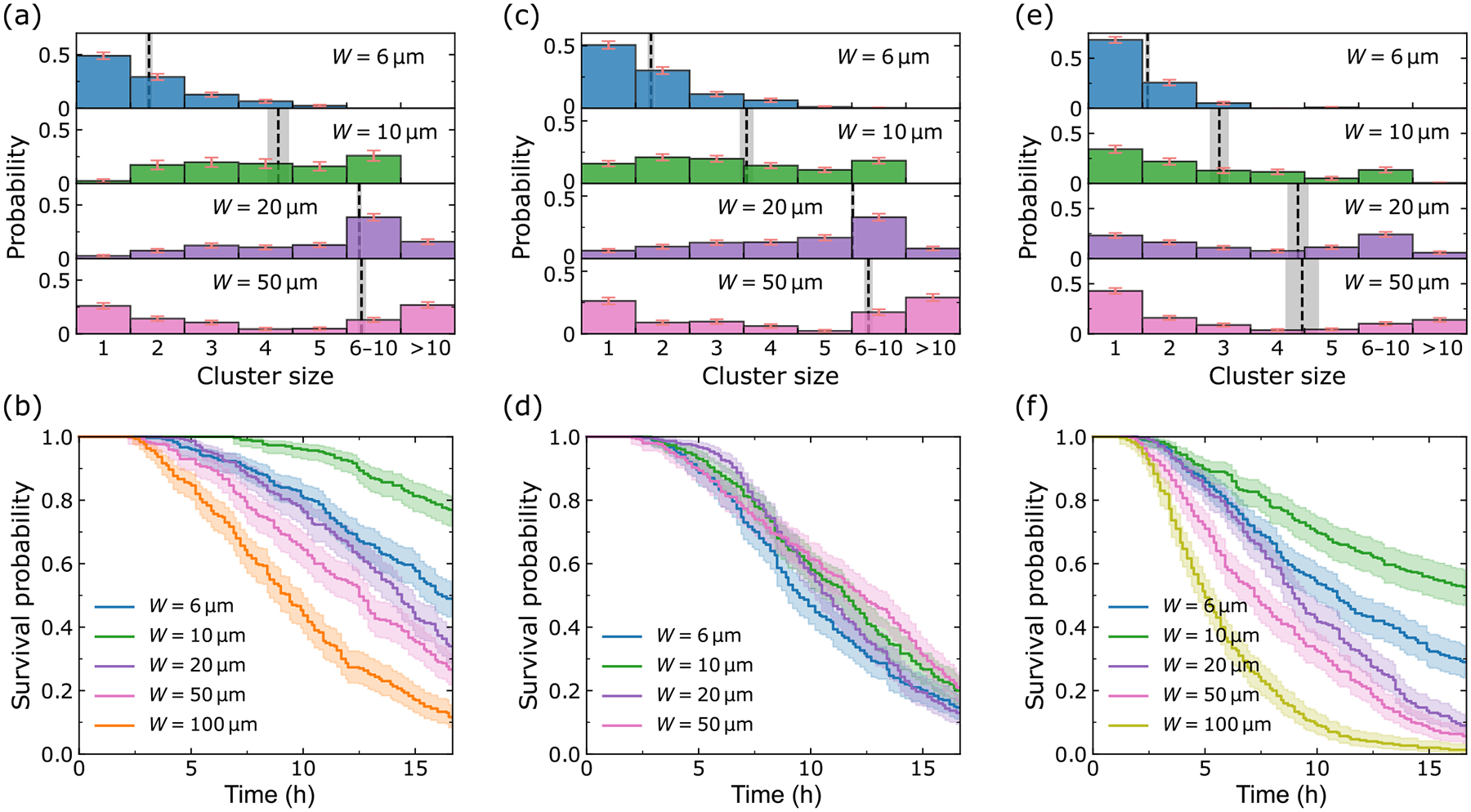
Simulation results for alternate models. Panels (a) and (b) are simulation results with neither leader cells nor tuning of cell-cell adhesion ($\omega =\omega_{0}$ in all microchannels). Panels (c) and (d) are simulation results after we tune down cell-cell adhesion in narrow microchannels (but no leader cells). Panels (e) and (f) are simulation results after we introduce the leader cells but still keep cell-cell adhesion $\omega =\omega_{0}$ in all microchannels. Three hundred independent simulations; chemotaxis strength $k_{\theta }=1.4k_{\theta }^{0}$. For the simulation results of 100-μm channels shown in panels (b) and (f), the total number of cells N is increased to 72.

**FIG. 11.**
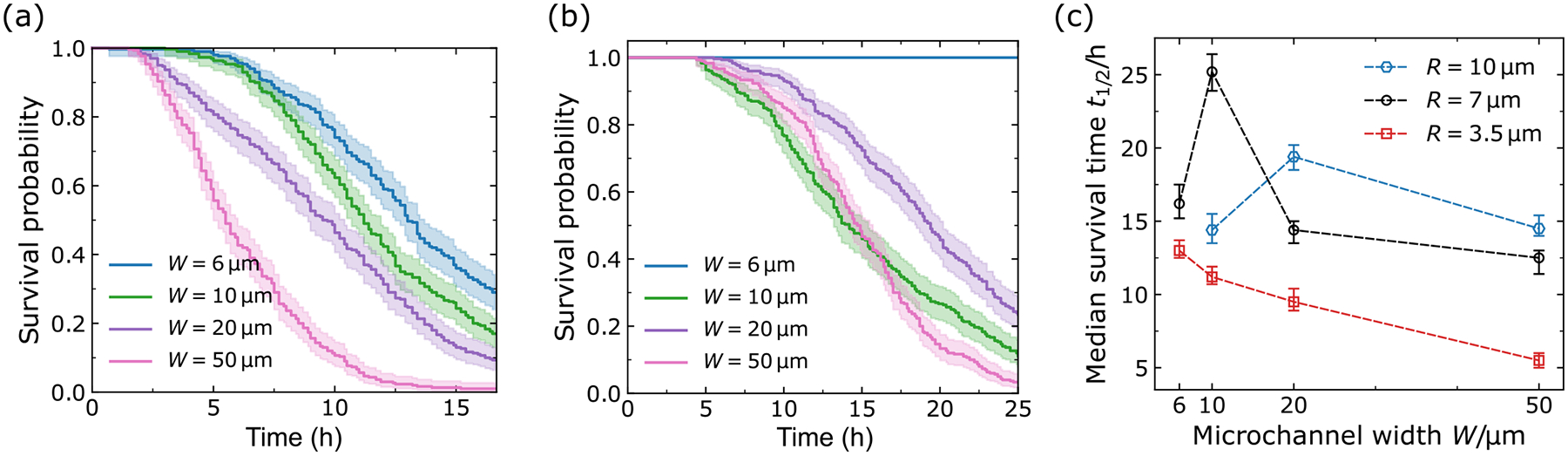
Simulation results for different cell sizes without special tunings (300 independent simulations, $\omega =\omega_{0}$ in all microchannels, $k_{\theta }=1.4k_{\theta }^{0}$, no leader cells). Panels (a) and (b) show the survival curves for different channel widths when the cell radius R=3.5 and 10μm, respectively. We notice that the 6-micron channels are too narrow for cells with a radius of 10μm to enter, resulting in no ruptures occurring [blue curve in panel (b)]. Panel (c) shows the median survival time $t_{1/2}$ as a function of microchannel width W for different cell sizes; the results for R=7μm (black) are extracted from Fig. 10(b); the error bars indicate the 95% confidence intervals. When changing the cell radius R to 3.5 and 10μm, we proportionally adjust the interfacial thickness $d=\sqrt{2K/\alpha }$ by varying α to fix d/R, and we also modify the number of cells N to 96 and 30 accordingly.

**FIG. 12.**
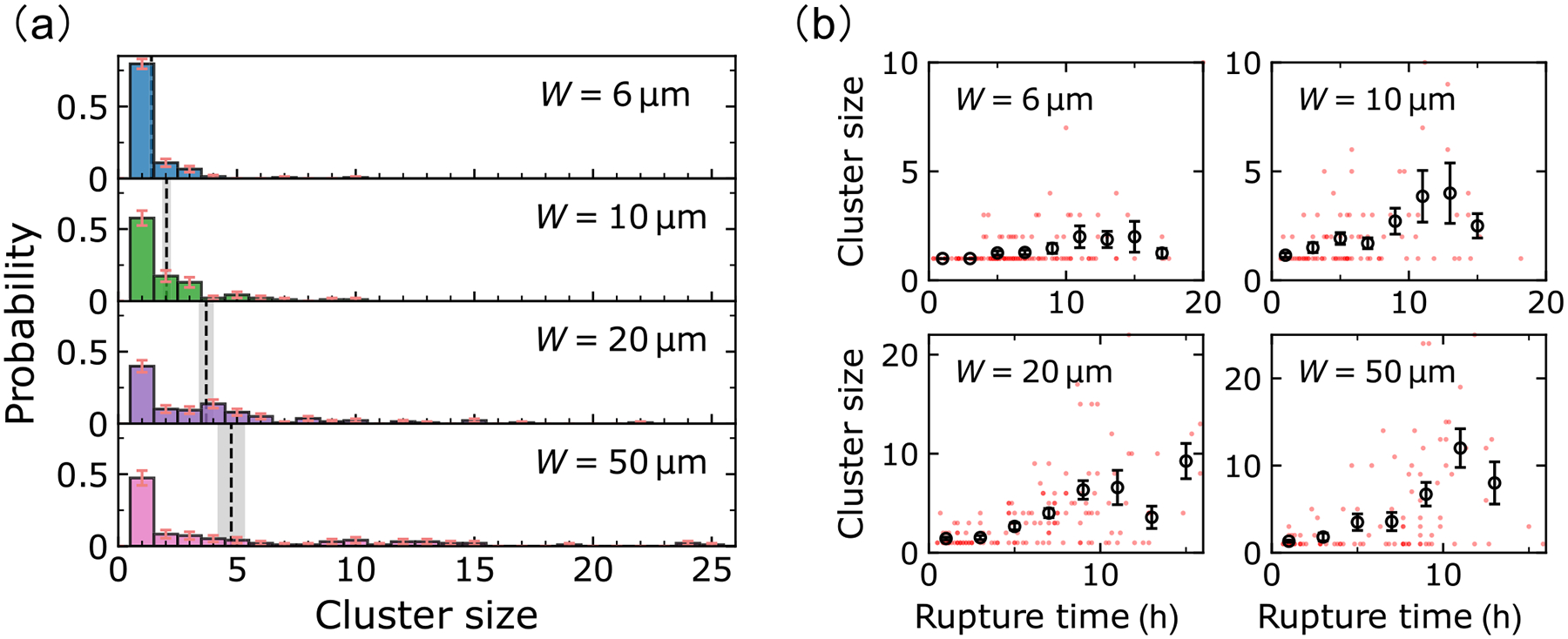
Experimental results. (a) Cluster-size distributions for microchannels of different widths. The dashed lines and gray areas denote the average cluster sizes A and their corresponding standard errors of the mean (SEM, or just SE). The error bars of the probabilities are determined by the binomial interval formula $\pm \sqrt{p(1-p)/n}$. (b) Scatter plots exhibit the (rupture time, cluster size) phase plane. The circles represent the average cluster sizes within each time bin (2 h), and the error bars indicate the corresponding SE. The standard error of the mean (SE) is calculated as $\text{SE}=\text{SD}/\sqrt{n}$, where SD is the standard deviation and n is the sample size.

**FIG. 13.**
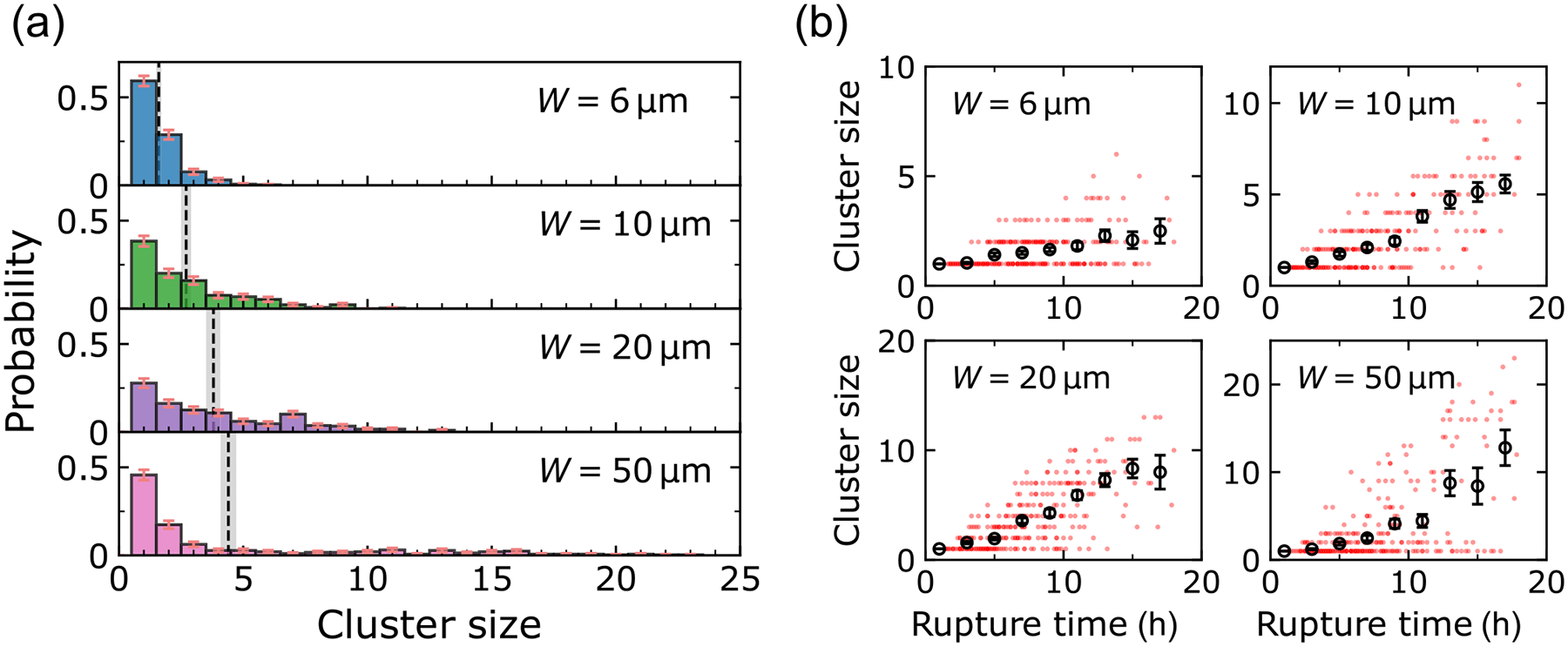
Simulation results with $\omega =0.7\omega_{0}$ for narrow channels, $\omega =0.8\omega_{0}$ for 20-μm channels, and $\omega =\omega_{0}$ for 50-μm channels. (a) Cluster-size distributions for different channel widths. The dashed lines and gray areas denote the average cluster sizes A and their corresponding standard errors of the mean (SE). Panel (b) displays the (rupture time, cluster size) phase plane scatter plots. The circles represent the average cluster sizes within each time bin (2 h), and the error bars indicate the corresponding SE.

Does the jamming transition influence the presence of rupture? To characterize the extent of jamming, we follow Ref. [7] and measure the effective diffusivity of cells as the order parameter, ${\overset{‾}{D}}_{\text{eff}}={\text{lim}}_{t\to \infty }\text{MSD}(t)/4D_{0}t$, where MSD(t) is the mean-squared displacement of the cells (see Appendix D). This is similar to the experimental quantification of jamming by, e.g., Ref. [54]. Here $D_{0}=p_{0}^{2}/2D_{r}$ is the diffusion coefficient for an isolated follower cell [7,8]. We control jamming by varying cell-cell adhesion and cell speed $p_{0}$. To compare with previous work, we characterize the cell’s speed by computing the dimensionless Péclet number $\text{Pe}=p_{0}/(RD_{r})$ [7], which is roughly speaking the distance an isolated cell would travel in the time the cell takes to reorient $\left(1/D_{r}\right)$, scaled by its size R. We measure the effective diffusivity of cells in the rectangular confinement (with periodic boundary condition in the x direction, see Fig. 8) of our previous simulations—but do not open the channel entrance, so all cells are confined in the original reservoir and there is no invasion. As shown in Fig. 9(a), we see a transition between near-ballistic motion at short timescales to more-diffusive motion at long times $\left(t>1/D_{r}\right)$. Mean-squared displacements are naturally larger for cells with larger motility $p_{0}$ (larger Pe). Fitting to a linear function to extrapolate to measure ${\overset{‾}{D}}_{\text{eff}}$, we can see that there is a clear transition in ${\overset{‾}{D}}_{\text{eff}}$ representing a fluid-solid transition [Fig. 9(b)]. Cells with low motility (the first two data points below the red dashed line) are in the solidlike jammed state, and cells with high motility (the rest points above the red dashed line) are in the unjammed state, which is consistent with Refs. [7,8]. The red dashed line is the threshold value 0.012, which we use to indicate the presence of a solid-fluid transition. This value is chosen both based on the sudden transition in Fig. 9(b) but also on the appearance of simulation movies showing the degree of cell rearrangement. Simulation snapshots for a typical jammed state and unjammed state are given in Fig. 8. We see that cells in the jammed state are highly ordered and near-crystalline, while the unjammed state shows more elongated, disordered cell shapes, as in Ref. [7].

To study the relationship between this jamming transition and our dissociation statistics, we run simulations of ruptures in 50-micron channels using the same procedure we used for Fig. 5 but varying the Péclet number. While we are varying the motility Pe, we keep R and $D_{r}$ constant and change the basal polarity magnitude to $p_{0}=\text{Pe}\cdot RD_{r}$. However, we still maintain the relationships $p_{\text{guided}}=2p_{0}$ and $p_{\text{leader}}=3p_{0}$, i.e., we are changing the self-propulsion of all cells in the system. Figure 9(c) shows there is no rupture for a system with $p_{0}$ at its two lowest values—when our jamming simulations indicate the tissue is in a solid state—and ruptures start to happen after the system is fluidized by increasing cell motility.

Is this a coincidence, or is the jamming transition always aligned with the rupture transition? We sweep the (ω, Pe) parameter plane to construct several phase diagrams, as shown in Figs. 9(d)–9(f). Both Figs. 9(d) and 9(e) show the rupture transition and that ruptures are completely abolished when the tissue has strong cell-cell adhesion but lower motility (red-point regions in the bottom right). We also notice—consistently with our earlier results—that larger ruptures tend to happen in a system of cells with strong cell-cell adhesion and high motility [Fig. 9(d), upper right corner].

We also measure jamming as a function of adhesion and motility in Fig. 9(f). Consistent with prior research findings [7,8], we have observed that a jammed state can be transitioned to an unjammed state by elevating cell motility. Furthermore, we also find the reinforcement of cell-cell adhesion will suppress unjamming, which is supported by experiments [14,58].

On comparing Fig. 9(f) to Figs. 9(d) and 9(e), it is evident that the red-point region indicating the solid states in Fig. 9(f) is a subset of the red-point region corresponding to no rupture in Figs. 9(d) and 9(e). In other words, unjamming alone does not ensure rupture—it is possible to have cells that are unable to invade a channel and rupture cell-cell adhesions (e.g., owing to low motility) but still have sufficient motility to fluidize the tissue. This includes, e.g., the points located in the lower center region of the (ω, Pe) plane. Therefore, we conclude that unjamming is necessary but not sufficient to create ruptures.

## V. DISCUSSION

To investigate the mechanism of how cells dissociate from collectively migrating strands in confinement, we reanalyzed data from our previous experiments [19] and used these results to motivate a phase-field simulation of invasion. We found in our experimental data that while larger channels led to larger cluster sizes, the most common outcome was always a single-cell dissociation event. To reproduce this effect within our model, we had to introduce the possibility that cells at the invasive front might spontaneously develop a leader state with a stronger-self-propulsion. Experimental measurements found no key difference between the time to rupture (survival time curves) for different channel sizes—opposite to the naive expectation that ruptures should be more common in channels with more cells. We were able to explain this feature of the data by assuming the cell adhesion weakens slightly in narrower channels relative to wider channels—a decrease of 30% in the narrowest channels. This was motivated by earlier results which find that contractility increases (leading to E-cadherin disengagement) and adhesions are more dynamic in confinement [19,43,44], but our results show that these effects need not be quantitatively dramatic to explain relatively large changes in rupture rates. Because explaining the survival curve in particular required this tuning of adhesion strength, we view this tuning as a prediction that could be validated by simultaneous measurement of cell-cell adhesion strength and rupture rates—though measuring cell-cell adhesion strength in the context of a narrow channel will likely be very experimentally difficult. We also identify key control factors within our model, showing that increases in cell-cell adhesion strength suppress rupture (as seen experimentally [19]), that the probability of cells becoming leaders changes the distribution of cluster sizes, and that increasing cell-wall adhesion can both permit rupture by permitting cells to invade into narrow channels, as well as suppressing it, by preventing cells from detaching from the wall.

For simplicity, in our current model, we have not included cell division and cell death. We think this is reasonable, given the timescale of our study (18 h)—we believe cells are primarily being driven by migration. However, division could quantitatively change the jamming phase diagram we find here, e.g., via changing fluidity [59,60] or by effects on cell polarity, speed, and cell-cell adhesion [61].

Earlier work has broadly argued that the degree of jamming and the ability of the tumor cells to rearrange may reflect whether cells from that tumor will metastasize [44,62,63]. We study, within our model, both the jamming transition and the rupture transition and find that unjamming of the tissue is *necessary*, but not sufficient, to predict rupture—there are many parameter sets where tissues are fluid but cancer cells are unable to dissociate. The details of this result may depend on assumptions of our model about cell-cell adhesion and motility. Researchers have employed various models to investigate the unjamming transition in tissues as a function of different control variables [8,9,24,54]. For example, studies using the vertex model [54] and the Voronoi model [8] have explored how cell shape index and motility influence the jamming transition. These models make predictions about the influence of cell-cell adhesion but adhesion enters only as an effective interfacial tension term [8,64], changing the ability of cells to deform, but not immediately penalizing cell-cell separation or sliding of cell-cell junctions. Our focus on nonconfluent tissues forces us to include cell-cell adhesion in a different form. We find that, in our phase-field model, increasing cell-cell adhesion leads to less rearrangement and increased jamming. However, even within phase-field models, there is no universal form of the cell-cell adhesion model, with Refs. [9,23,24,30] all including adhesion in slightly different forms, and Ref. [7] disregarding it entirely, while Ref. [10] does not include a cell-cell adhesive force but does include the effect of a cell-cell friction, which also changes dynamics of rearrangement. Our model could also be extended to include more explicit dynamics of bond rupture [47,65,66]. Broadly, our results that increasing cell-cell adhesion leads to jamming is supported by experiments but with some contradictory results. Many experiments support the idea that decreasing cell-cell adhesion can lead to tissue fluidization [14,58,67,68] consistent with our observation. However, unjamming may also co-occur with the amplification of local adhesive inter-cellular stresses in human bronchial epithelial cells [54].

Our work shows that increasing cell-cell adhesion strength decreases the rate of rupture, but—if that rupture happens—increases the average size of the cluster that dissociates. This result may be relevant to the larger re-evaluation of the role of E-cadherin in cancer. The loss of intercellular adhesion molecule E-cadherin has long been considered a hallmark when tumor cells transition into an invasive phenotype resulting in cancer metastasis [69]. However, recent experiments have shown that E-cadherin expression is paradoxically correlated with cancer metastasis—while loss of E-cadherin increases invasion, it also reduces cancer cell proliferation and survival rates [69,70]. Larger dissociation events may also be an aspect of this puzzle, because large-cluster dissociations may be more successful metastases [5].

We note that in our simulations at default parameters, all the survival probabilities drop to almost 0 [Fig. 5(b)], while the experimental survival curves saturate to approximately 30% [Fig. 2(b)]. The saturation of the experimental survival curves means either that there is something special about 30% of experiments (e.g., high degree of variability between channels) or that the system is aging—i.e., the probability of dissociation must decrease sharply as a function of time. There are two known sources of aging. First, the chemical gradient in the experiments of Ref. [19] is created by simple diffusion and will become more shallow over time, potentially decreasing the strength of chemotaxis, though we do not expect this to be relevant in the most narrow channels. Decreasing chemotactic strength leads to the flattening of survival curves in our model [Fig. 7(d)]. However, this is a weaker effect and does not completely suppress dissociation. We view it as more likely that cell-cell adhesion strength increases over the experiment time, as has been previously observed in epithelial jamming [71]. Increasing cell-cell adhesion can completely suppress dissociations (Fig. 9). The survival of a small percentage of channels could also potentially reflect channel-to-channel variability in the experiment, e.g., in geometry or fibronectin coating. In a trivial example, if 30% of channels were not adherent, then there would be no invasion in these channels—and hence no dissociations. While we do not observe anything this dramatic, variability of this sort (or day-to-day variability [72]) may represent additional potential confounders.

Our model simply assumes the polarity of cells follows a random reorientation, biased by the chemoattractant gradient [Eq. (3)]. This neglects any possible coordination of cell polarities between neighboring cells, e.g., alignment of cell polarity to neighbors [73], contact inhibition of locomotion (CIL) leading to repolarization [23,31,74], or many others reviewed in Ref. [75]. We view our assumptions here as a first theoretical approach, but careful tests of cell-cell interaction may require us to extend our model. For instance, Ref. [37] shows that relatively rare populations of invasive leader cells can drive the invasion of nonleader cells, which could potentially be captured by this sort of cell-cell guidance [12,76]. We also can address the possible roles of chemotaxis (directed cell migration) vs chemokinesis (chemically dependent increase in speed) in our simulations and experiments. We expect that the chemoattractant FBS is important both in terms of a chemokinetic factor and a chemoattractant. In the experiment, the absence of FBS results in significantly fewer dissociation events in 6-micron channels (see Fig. S3c from Ref. [19]), but changing gradient strength does not change dissociation. In our simulations, if there is no chemokinetic effect, i.e., if we reduce the cell velocities so that leader cells have the base self-propulsion strength $p_{0}$, as a model for the no-FBS condition, rupture is strongly suppressed. This no-FBS condition is similar to the point Pe ≈ 0.5 in the phase diagram of Fig. 9. The absence of chemotaxis but the presence of a chemokinetic factor (modeling constant but high FBS concentration) can be modeled by setting $k_{\theta }=0$ in the model—removing any directed migration. Our results in Figs. 7(c) and 7(d) for very small $k_{\theta }$ indicate that cells are less coordinated, which leads to smaller cluster sizes. However, the survival probability curves are less strongly affected by the absence of chemotaxis.

Our work has focused on the rupture and dissociation in collective cancer invasion, but it may have interesting connections to fracture events in other biological systems. Reproduction by fission in the metazoan *Trichoplax adhaerens* occurs by a motility-driven ductile-to-brittle transition of their epithelial layers [77], which might also reflect some of the control parameters we identify here. Fracture of epithelial monolayers under strain has been shown to depend on both keratin organization within the cell and cell-cell adhesive bond rupture [47]. Epithelial fracture has also been modeled within a vertex model with a detachment energy [48], predicting that the transition between the ability of the tissue to rearrange under strain and its tendency to break depends on the effective interfacial tension and contractility of the cells. Our model may also reflect this difference—but in our case, ruptures are not induced by a strain applied to the tissue but self-generated by the cells’ noisy motility. Formation of holes and voids can also occur in the spongy mesophyll, resulting in stable porous cell networks [78], or driven by cell contractility in epithelioid tissues [79]. An exciting future line of research is to understand to what extent and at what rate fracture events of all these various types can occur spontaneously, driven by randomness in cell motility and by chemical gradients, as observed in our work.

## Supplementary Material

SI movies (zipped)

## Figures and Tables

**FIG. 14.**
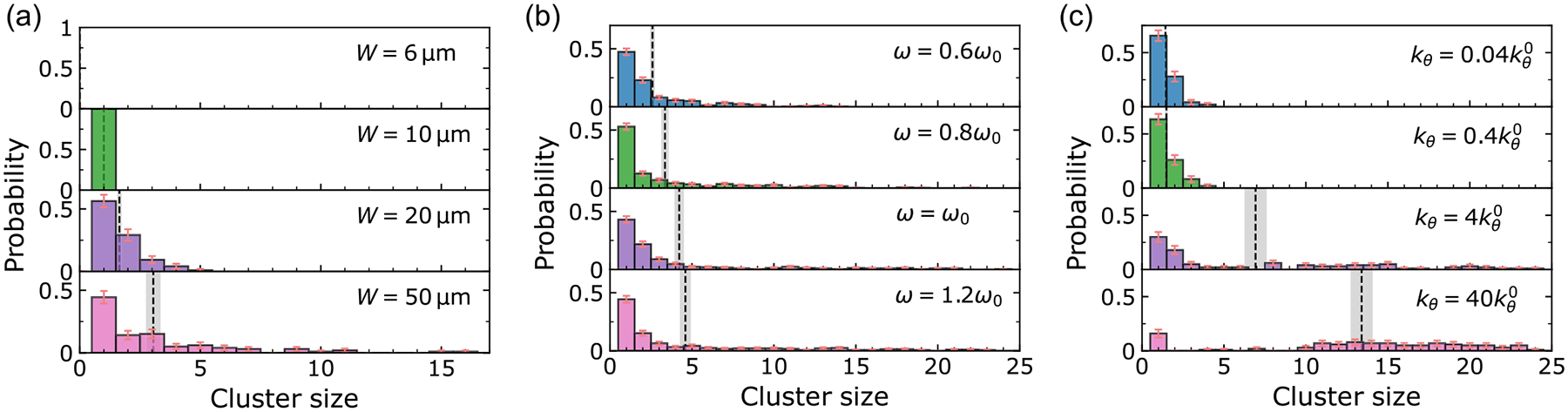
Simulation results. (a) Histograms when cell-wall adhesion κ is set to 0. (b) Histograms for different adhesion strength ω in 50-μm channels. (c) Histograms for different chemotaxis strength $k_{\theta }$ in 50-μm channels. The dashed lines and gray areas in (a)–(c) denote the average cluster sizes A and their corresponding SE.

**FIG. 15.**
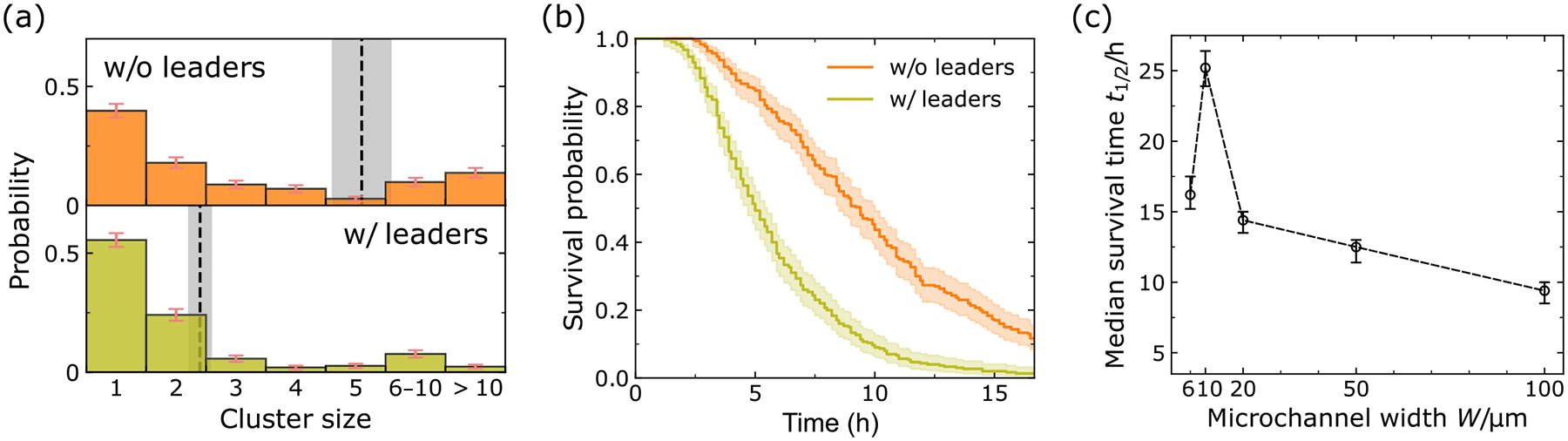
Simulation results for W=100-μm channels with cell-cell adhesion fixed at $\omega =\omega_{0}$. (a) Histograms for Model 1 (without leader cells) and Model 3 (with leader cells). (b) Survival curves for Model 1 and Model 3, which are also presented in Figs. 10(b) and 10(f). (c) Median survival time $t_{1/2}$ for R=7μm, extended to 100μm based on Fig. 11(c). Results are based on 300 independent simulations with the chemotaxis strength of $k_{\theta }=1.4k_{\theta }^{0}$, and the number of cells N increased to 72.

## Data Availability

Code to reproduce this paper has been deposited at Zenodo [80].

## Footnotes

1 We note this force balance interpretation is not quite correct for our model, because the terms $\partial \phi_{i}/{\partial t|}_{\text{adh}}$ are not derived from a Hamiltonian.

## APPENDIX A: ALTERNATE MODELS

In this section, we present various models with different assumptions we have tested and show the process of how we reach our final model given in the main text.

### 1. Initial baseline model

As a starting point, we implement the simplest model with fixed intercellular adhesion $\omega =\omega_{0}$ and only two cell states. We designate cells inside the channel or near the channel entrance as guided cells, while all other cells are classified as follower cells. The statistical results of our simulations are shown in Figs. 10(a) and 10(b). We can see that the average cluster sizes are larger than those observed in the experiments, and single-cell ruptures are not predominant in all channels. Additionally, the survival curves decline much more slowly compared to the experiments and do not collapse. By intuition, we would expect the survival probability to decrease with increasing channel width. Surprisingly, we notice that the 10-μm curve is higher than the other curves. We will discuss this fact later in Appendix B.

### 2. Downregulating intercellular adhesion

In the experiments, we observe a good collapse of all survival curves for different channel widths on top of each other. However, the survival curves of our initial model in Fig. 10(b) do not collapse. We attempt to address this issue by reducing intercellular adhesion in narrow channels, as motivated by earlier experimental work [19]. With a reduction of only 30% in 6- and 10-micron channels and 20% in 20-micron channels, we achieve the collapse of all survival curves [see Fig. 10(d)], but the cluster-size distribution and the average cluster size [Fig. 10(c)] are still qualitatively different from the experimental data, with only rare single-cell ruptures.

### 3. Introducing leader cells

Compared to the initial model, here we introduce the high-metabolic-activity leader cells at the leading edge into the model but do not tune cell-cell adhesion as a function of channel width. We notice that single-cell ruptures are predominant now [see Fig. 10(e)]. As for the survival probability, they decrease more rapidly with time but still do not collapse onto a single curve [Fig. 10(f)]. Again, we see that the 10-micron channel has the longest survival curves, which we address in Appendix B.

### 4. Final model

Our final model featured in the main text combines the adjustments made in Models 2 and 3: downregulating inter-cellular adhesion in narrow channels and introducing leader cells, which recapitulates both the cluster-size distribution and the collapse of survival curves observed in experiments.

## APPENDIX B: HOW DOES CELL SIZE AFFECT RUPTURES?

By intuition, we would expect the survival probability to decrease with increasing channel width. However, as we can see in Figs. 10(b) and 10(f), the 10-micron channel has slower ruptures than other channels. The reasoning behind the slower ruptures in the 10-micron channel lies in the proximity of its size to the default cell size (preferred radius R=7μm) in our model. In the 10-micron channel, cells exhibit a broader cell-cell contact line compared to the 6-micron channel, making it harder to have ruptures—but in the 20-micron or wider channels, multiple cells can enter at once, so ruptures do not require breaking a larger contact line. Within our rough guiding hypothesis, we think of the 10-micron channel as having a higher initial energy barrier.

To verify our hypothesis, we first define the median survival time as the “half-life” of a survival curve S(t), i.e., the time when S(t)=0.5, or more explicitly,

(B1)
$$t_{1/2}=S^{-1}(0.5).$$

For the default R=7μm cells, we observe a clear peak of $t_{1/2}$ [extracted from Fig. 10(b)] at W=10μm in Fig. 11(c). If our hypothesis holds true, then the position of the peak should always be around the cell diameter 2R. Therefore, we next vary the preferred cell radius R to see how the peak of median survival time $t_{1/2}$ changes. Figures 11(a) and 11(b) present the survival probabilities of the initial model for R=3.5μm and R=10μm, respectively. We notice that in Fig. 11(b), the 6-micron channels are too narrow for the cells with a diameter of ~20μm to enter, resulting in the survival probability remaining at 1. After computing the corresponding median survival times, we find that the channel width where $t_{1/2}$ peaks is indeed around the cell diameter and increases with cell size [Fig. 11(c)], which confirms our thoughts.

## APPENDIX C: INITIAL CONDITIONS

We first consider the static solution to the equation of motion [Eq. (1)] for an isolated cell in one dimension ϕ(x), so the active polarity vector $P=0,{ℋ}_{\text{exclusion}}=0$, and all adhesion terms vanish. If the area of the cell is fixed, then the area deviation term can also be ignored. Thus Eq. (1) degenerates to $\delta {ℋ}_{\text{CH}}/\delta \phi =0$ now, and the corresponding Euler-Lagrange equation of ${ℋ}_{\text{CH}}$ is [26]:

(C1)
$$\frac{{\text{d}}^{2}\phi (x)}{\text{d}x^{2}}=\frac{\alpha }{K}[\phi^{3}(x)-\frac{3}{2}\phi^{2}(x)+\frac{1}{2}\phi (x)].$$

A domain wall can be introduced by forcing the two sides of the field to be in different phases, e.g., requiring ϕ(x→-∞)=0 and ϕ(x→∞)=1 as boundary conditions. By using the identity ${\text{d}}^{2}\text{tanh}(ax)/\text{d}x^{2}=-2a^{2}{\text{sech}}^{2}(ax)\text{tanh}(ax)$, it can be readily verified that the profile:

(C2)
$$\phi (x)=\frac{1}{2}+\frac{1}{2}\text{tanh}\left(\frac{x-x_{0}}{2d}\right),$$

is a solution to the above nonlinear differential equation [Eq. (C1)] adhering to the boundary conditions, provided $d=\sqrt{2K/\alpha }$ and $x_{0}$ represent the width and position of the domain wall. Since our phase-field model is in two dimensions, we use

(C3)
$$\phi (r)=\frac{1}{2}+\frac{1}{2}\text{tanh}\left(\frac{R-r}{2d}\right),$$

as the initial configuration, where $r=|r-r_{0}|$ is the distance to the center of the cell $r_{0}$. The geometric-confinement field $\phi_{\text{wall}}(r)$ is defined by a series of logistic sigmoid functions in a similar manner.

## APPENDIX D: NUMERICAL DETAILS

By evaluating the functional derivative of the total Hamiltonian [Eq. (2)], the equation of motion [Eq. (1)] can be written as

(D1)
$$\frac{\partial \phi_{i}}{\partial t}=-P_{i}\cdot \nabla \phi_{i}-\frac{1}{\gamma }[\alpha (\phi_{i}^{3}-\frac{3}{2}\phi_{i}^{2}+\frac{1}{2}\phi_{i})-K\nabla^{2}\phi_{i}\;-\frac{4\lambda \phi_{i}}{\pi R^{2}}\delta V[\phi_{i}]+2g\phi_{i}(h(r)-\phi_{i}^{2})+2g_{\text{wall}}\phi_{i}\phi_{\text{wall}}^{2}]\;-\sum_{j\neq i}\omega f(\nabla \phi_{j})\cdot \nabla \phi_{i}-\kappa f(\nabla \phi_{\text{wall}})\cdot \nabla \phi_{i},$$

where we have introduced another auxiliary field $h(r)=\sum_{i}\phi_{i}^{2}(r)$ for parallel computing purpose (the simulation C++ code is parallelized on multiprocessors by Open MPI) [7]. The area deviation term is $\delta V[\phi_{i}]=1-\int {\text{d}}^{2}r\phi_{i}^{2}/(\pi R^{2})$, and we normalize the gradient via a saturation function $f(\zeta )=\zeta /\sqrt{1+\epsilon |\zeta {|}^{2}}$ to prevent numerical instability resulting from very steep gradient [24]. Then, we employ forward differences to evolve the phase-field equation for each cell, i.e.,

(D2a)
$$\phi_{i}(t+\Delta t)=\phi_{i}(t)+\text{R.H.S.}(t)\times \Delta t,$$

(D2b)
$$\theta_{i}(t+\Delta t)=\theta_{i}(t)-k_{\theta }[\theta_{i}(t)-\theta_{0}]\times \Delta t+\Theta ,$$

where R.H.S.(t) is the right-hand side of Eq. (D1), the noise $\Theta \sim 𝒩(0,2D_{r}\Delta t)$, and the preferred direction $\theta_{0}$ points toward or along channels $\left(\theta_{0}=\pi /2\right)$. When computing the gradient ∇ϕ and Laplacian $\nabla^{2}\phi$, we apply first- and second-order central differences, respectively. We have matched all default parameter values in our model with the experiments, which are given in Table I.

During the simulation, we first implement a “thermalization” process for $T_{\text{therm}}=5\;\text{h}$ which allows all cells to evolve from the initial conditions we imposed and reach more natural shapes. After this process, we open the channels and keep on watching for $T_{\text{tot}}=18\;\text{h}$, consistent with the experiments. Our study focuses on the first dissociation event within the channel. Therefore, we terminate the simulations once the first dissociation occurs for each channel, recording the rupture times and rupture sizes for statistical analysis. (The only exception to this is the simulations in Fig. 4, which we run to show the separation dynamics of the clusters as an illustration.) As a result, reattachment following dissociation is not considered in our analysis. Note that while we implement periodic boundary conditions in both the x and y directions, there is also a bottom wall, making the channels effectively closed at the top. We expect that the presence of this wall does not influence our core statistical results on dissociations. In our simulations, ruptures generally occur before cells hit the wall, and all channels generally have a rupture (Fig. 5)—except when motility is slowed (low Pe), in which case cells do not even reach the end of the channel. Once the first cells of the invading strand cells hit the wall, this suppresses dissociation—similar to if we had ended the simulation when cells impact the wall or removed them from the system, the other plausible boundary conditions. However, the presence of a wall (like the presence of a finite channel size in the experiments of Ref. [19]) could impact the very long-time dynamics in cases when dissociation is slow. Our simulated system size (channel length of 200 microns) is slightly smaller than the experimental channels (~400 microns); this effect, like our finite number of cells, could in principle reduce the likelihood of extremely large clusters being found, though we have seen no evidence of this in the experiment.

The initial magnitudes of polarity for all cells are set to $p_{0}$. Cells become guided once they get close enough to the channel. We define a box of (W+24μm)×6μm at the channel entrance in the simulation. Once the center of mass of a cell crosses into this region or inside the channel, the cell is considered a guided cell $\left(k_{\theta }\neq 0\right)$, which can sense the chemical gradient and move with a larger velocity (magnitude of polarity set to $2p_{0}$). We also introduced leader cells at the invading front in our model—cells have a probability of becoming leader cells when they reach the front (become a tip cell). First, we have to define what a tip cell is as follows: If there is any point of the cell surface where there is not a cell in the monolayer immediately above it, then it is at the tip. In our model, whenever a guided cell becomes a tip cell, there is a probability for it to transition into a leader (magnitude of polarity set to $3p_{0}$). We give a sketch of the algorithm for this process in Algorithm 1. As discussed in the main text, the probability of becoming a leader cell inversely scales with its contact length with other cells in our model—cells are more likely to become leader cells if they have a lot of free space. Based on the form of the adhesion term in the model, we count a grid point at position r as a contact point of cell i when $|\nabla \phi_{i}(r)\cdot \sum_{j\neq i}\nabla \phi_{j}(r)|=|\nabla \phi_{i}\cdot (\nabla h-\nabla \phi_{i})|>0.01\;\mu {\text{m}}^{-2}$. Therefore, Eq. (4) is calculated as $P_{i}^{\text{leader}}=\text{max}(1-n_{i}/n_{c},0)$ in practice, where $n_{i}$ is the number of contact points of cell i, and $n_{c}$ is set to 256 in our simulation by default. Also, we note that in Algorithm 1 when a leader cell is no longer at the tip, it will revert to a guided cell state.

**TABLE I.** Table of simulation parameters^a^.

Parameter	Description	Dimension	Dimensionless value	Real value
δx	Lattice constant	L	1	1μm
δt	Simulation time unit	T	1	15 s
N	Number of cells	1	48	48
Δt	Simulation time step	T	0.4	6 s
R	Target cell radius	L	7	7μm
$p_{0}$	Advection velocity (polarity)	L/T	5.556 × 10^−2^	13.3μm/h
$D_{r}^{-1}$	Reciprocal of the rotational diffusion coefficient	T	180	45 min
d	Interfacial thickness	L	2	2μm
γ	Friction coefficient	$ET/L^{2}$	12	
K	Cell tension coefficient	E	3.5	
α	Cell interfacial coefficient (defined as $2K/d^{2}$)	$E/L^{2}$	1.75	
λ	Cell area coefficient	E	250	
g	Cell-cell repulsion coefficient	$E/L^{2}$	4	
$g_{\text{wall}}$	Cell-wall repulsion coefficient	$E/L^{2}$	5	
ω	Cell-cell adhesion coefficient	$L^{2}/T$	3.5–5	
$\omega_{0}$	Reference value of adhesion coefficient	$L^{2}/T$	5	$\sim 0.3\;{\mu \text{m}}^{2}/\text{s}$
κ	Cell-wall adhesion coefficient	$L^{2}/T$	3.5	$0.23\;\mu {\text{m}}^{2}/\text{s}$
ϵ	Regularization coefficient	$L^{2}$	37.25	$37.25\;{\mu \text{m}}^{2}$
$k_{\theta }$	Chemotaxis strength coefficient	1/T	$10^{-3}-0.05$	$0.004-0.2\;{\text{min}}^{-1}$
$k_{\theta }^{0}$	Reference value of chemotaxis strength coefficient	1/T	0.0025	$0.01\;{\text{min}}^{-1}$
$n_{c}^{0}$	Default characteristic number of contact points $n_{c}$	1	256	

a These parameters are used throughout the paper; any deviation from them is explicitly noted.

To improve computational efficiency, we also implement domain decomposition [7,22,23,28], where we solve for each $\phi_{i}$ only within a smaller subdomain that shifts with the center-of-mass $R_{i}^{\text{c.m.}}$ of each cell. The center-of-mass position for each cell is computed using the following equation:

(D3)
$$R_{i}^{\text{c.m.}}=\frac{1}{M_{i}}\int {\text{d}}^{2}rr\phi_{i}(r),$$

where $M_{i}:=\int {\text{d}}^{2}r\phi_{i}(r)$ is the total “mass” of the cell. Thus the center-of-mass velocity is readily given by $v_{i}^{\text{c.m.}}=\text{d}R_{i}^{\text{c.m.}}/\text{d}t$. The mean-squared displacement mentioned in Sec. IV G is computed by [7]

(D4)
$$\text{MSD}(t)={\left\langle \frac{1}{N}\sum_{i=1}^{N}{\left[R_{i}^{\text{c.m.}}(t+\tau )-R_{i}^{\text{c.m.}}(\tau )\right]}^{2}\right\rangle }_{\tau },$$

where the average ⟨⋯⟩ is taken over time $\tau \in [0,T_{\text{tot}}-t]$.

We apply standard Kaplan-Meier survival analysis [21] to handle the data and generate survival probability plots using the lifelines.KaplanMeierFitter class provided by the Python survival-analysis package lifelines [81]. The 95% confidence intervals are computed using the exponential Greenwood confidence interval method [82].

## APPENDIX E: SUPPLEMENTAL SIMULATION MOVIE CAPTIONS

*Movie 1:* Dissociations of collectively invading monolayers in microchannel of W=6μm. Snapshots of this movie are presented in Fig. 4. All parameters are set to default values. Scale bar, 15μm.

*Movie 2:* Dissociations of collectively invading monolayers in microchannel of W=10μm. Snapshots of this movie are presented in Fig. 4. All parameters are set to default values. Scale bar, 15μm.

*Movie 3:* Dissociations of collectively invading monolayers in microchannel of W=20μm. Snapshots of this movie are presented in Fig. 4. All parameters are set to default values. Scale bar, 15μm.

*Movie 4:* Dissociations of collectively invading monolayers in microchannel of W=50μm. Snapshots of this movie are presented in Fig. 4. All parameters are set to default values. Scale bar, 15μm.

*Movie 5:* Cells can not enter the narrow microchannel without cell-wall adhesion. Microchannel width W=6μm. All parameters are set to default values except the cell-wall adhesion strength κ=0. Scale bar, 15μm.

*Movie 6:* Cells in jammed state. Parameters are default except Péclet numbers is Pe = 0.14. Scale bar, 15μm. Snapshots of this movie are presented in Fig. 8.

*Movie 7:* Cells in unjammed state. Parameters are default with Péclet numbers Pe = 1.43. Scale bar, 15μm. Snapshots of this movie are presented in Fig. 8.

## APPENDIX F: ADDITIONAL FIGURES

Within the main paper (e.g., Figs. 2 and 5), we show the cluster size histograms with some cluster sizes grouped together, for legibility and clarity. In this section, we provide the full histograms (Figs. 12–14). We also show the scatter plots of cluster size vs rupture time for different channel widths. In addition, we provide some results on larger channels (Fig. 15).
